# Metagenomic analysis of microbial consortia enriched from compost: new insights into the role of Actinobacteria in lignocellulose decomposition

**DOI:** 10.1186/s13068-016-0440-2

**Published:** 2016-01-29

**Authors:** Cheng Wang, Da Dong, Haoshu Wang, Karin Müller, Yong Qin, Hailong Wang, Weixiang Wu

**Affiliations:** Zhejiang Province Key Laboratory for Water Pollution Control and Environmental Safety Technology, Institute of Environmental Science and Technology, Zhejiang University, 866 Yuhangtang Road, Hangzhou, 310058 China; Ruakura Research Centre, The New Zealand Institute for Plant and Food Research Limited, Private Bag 3123, Hamilton, New Zealand; Key Laboratory of Soil Contamination Bioremediation of Zhejiang Province, School of Environmental and Resource Sciences, Zhejiang A & F University, Lin’an, Hangzhou, 311300 China

**Keywords:** Lignocellulose degradation, Metagenomics, Compost ecosystem, Actinobacteria

## Abstract

**Background:**

Compost habitats sustain a vast ensemble of microbes specializing in the degradation of lignocellulosic plant materials and are thus important both for their roles in the global carbon cycle and as potential sources of biochemical catalysts for advanced biofuels production. Studies have revealed substantial diversity in compost microbiomes, yet how this diversity relates to functions and even to the genes encoding lignocellulolytic enzymes remains obscure. Here, we used a metagenomic analysis of the rice straw-adapted (RSA) microbial consortia enriched from compost ecosystems to decipher the systematic and functional contexts within such a distinctive microbiome.

**Results:**

Analyses of the 16S pyrotag library and 5 Gbp of metagenomic sequence showed that the phylum Actinobacteria was the predominant group among the Bacteria in the RSA consortia, followed by Proteobacteria, Firmicutes, Chloroflexi, and Bacteroidetes. The CAZymes profile revealed that CAZyme genes in the RSA consortia were also widely distributed within these bacterial phyla. Strikingly, about 46.1 % of CAZyme genes were from actinomycetal communities, which harbored a substantially expanded catalog of the cellobiohydrolase, β-glucosidase, acetyl xylan esterase, arabinofuranosidase, pectin lyase, and ligninase genes. Among these communities, a variety of previously unrecognized species was found, which reveals a greater ecological functional diversity of thermophilic Actinobacteria than previously assumed.

**Conclusion:**

These data underline the pivotal role of thermophilic Actinobacteria in lignocellulose biodegradation processes in the compost habitat. Besides revealing a new benchmark for microbial enzymatic deconstruction of lignocelluloses, the results suggest that actinomycetes found in compost ecosystems are potential candidates for mining efficient lignocellulosic enzymes in the biofuel industry.

**Electronic supplementary material:**

The online version of this article (doi:10.1186/s13068-016-0440-2) contains supplementary material, which is available to authorized users.

## Background

The dramatic rate of fossil fuels depletion and the dwindling supplies of geological reserves have spurred the search for alternative renewable energy sources such as biofuels. Utilization of lignocellulosic biomass derived from renewable plant material represents a promising alternative to fossil fuel [1]. However, the effective conversion of plant biomass remains a formidable challenge due to the paucity of highly efficient enzymes. Furthermore, most of the attractive lignocellulosic biomass conversion strategies are insensitive to fluctuations in feedstock and frequently dependent on thermal or chemical pre-treatments of the biomass [2]. As a consequence, search for microbes and enzymes displaying these desired characteristics of stability and efficiency in habitats where lignocellulosic biomass is naturally processed by microbiomes seems promising.

To recycle organic waste sustainably, composting is generally mediated by indigenous microbial communities under the aerobic conditions and in the solid state. Given that composts sustain an immense diversity of microbes for the efficient degradation of lignocellulosic biomass [3, 4], compost habitats are considered as one of the most important bioreactors for renewable bioenergy on the planet [5]. Therefore, various research groups have attempted to characterize these functional microbial communities. A range of thermophilic cellulose-degrading bacteria including, for example, *Bacillus* spp., *Thermus* spp., and *Streptomycetes* spp. have been isolated from compost and cultured in laboratory studies [6]. Moreover, many culture-independent molecular analyses with PCR-DGGE and clone libraries observed that cellulolytic actinomycetes are enriched in compost habitats. These observations suggest that thermophilic *Saccharomonospora viridis* and *Thermobifida fusca* as well as mesophilic *Micrococcineae* jointly contribute to the degradation of lignocellulosic materials [7, 8]. However, because of the complexity of lignocellulosic enzyme systems and compost microbiomes, it has generally been considered unfeasible to characterize the microbial degradation of lignocellulose in compost ecosystems by applying the above-mentioned techniques, which are principally based on isolated pure strains or 16S rRNA gene cloning. This not only inhibits furthering our understanding of the functions, interactions and ecology of microbial communities related to the degradation and transformation of lignocellulosic materials, but also seriously impedes exploiting the repertoire of novel enzymes and proteins that could enhance the efficiency of biomass conversion in compost ecosystems.

To date, the development to exploit ‘-omics’ approaches for the identification of the metabolic functions of multispecies consortia has progressed considerably [9]. These powerful approaches allow the identification of lignocellulosic enzymes and the resulting comprehensive sets of translated gene sequences can be used to infer the physiology and biochemistry of lignocellulose degradation processes. Recent advances in metagenomics generated functional genomic datasets of diverse environmental samples, including bovine rumen [10], guts of termite, panda, and honey bee [11–13], actively fermenting platform communities and natural grassland soil [14, 15]. These studies suggest that metagenomics approaches could provide an overview of the functioning of environmental microbial communities. Furthermore, such metagenomics approaches revealed the cellulolytic capabilities of various complex microbiome samples and substantially expanded the repertoire of genes and genomes participating in the decomposition of lignocellulose, which are of interest for industrial biorefineries and white biotechnology. To the best of our knowledge, to day a few metagenomic analyses have been conducted to analyze the structure and potential function of microbial communities in the compost ecosystem, which is also regarded as a unique and highly efficient system for bio-recycling of lignocellulosic biomass [5, 16]. However, the mechanism by which resident microbial communities synergistically degrade and utilize the lignocellulosic plant biomass under high temperature and solid conditions of the compost habitat is poorly defined.

It is almost universally accepted that the enrichment culture technique is a powerful tool for building microbial consortia with desired properties [17, 18]. A typical enrichment method for generating a desired consortium is to adapt environmental communities to feedstocks under specific conditions, with the expectation that the developing communities will be enriched with microbes displaying the desired functions. For example, Hess et al. [9] successfully obtained an enriched fiber-degrading microbial community on switchgrass incubated within the rumen. Likely, DeAngelis et al. [19] demonstrated that the switchgrass-adapted communities were less complex and more suited to degrading targeted feedstock than the starting inoculum. It should also be noted that the enrichment technique could lower the complexity of metagenomic datasets, subsequently rendering its future assembling more amenable [19].

Hence, in this study, a unique rice straw-adapted (RSA) microbial community was enriched within a nylon bag throughout the cow manure composting process. Owing to the intensive degradation of lignocellulosic biomass occurring during the thermophilic and maturing stages of composting process [7, 20, 21], the nylon bag filled with rice straw was retrieved at the end of maturing stage that is suggested to be the optimal time to fully explore the large repertoire of microbes and genes participating in the lignocellulose decomposition within the compost habitat. We therefore applied 454 pyrosequencing and massive depth metagenomic sequencing to systematically complement the microbial taxonomic analyses and to characterize the microbial functional diversity of the RSA consortia. The specific investigation of carbohydrate-active enzymes repertoire and their phylogenetic distribution in the RSA consortia will contribute to a better understanding of the microbial enzymatic decomposition of lignocelluloses in the compost ecosystem with extreme complexity. Ultimately, this study will provide valuable insights into the compost microbiome for bioprospecting novel lignocellulosic biocatalysts in the biofuel industry.

## Results and discussion

### Changes in the pre- and post-enrichment rice straw contents and composition

After 30 days of enrichment, substantial degradation of the rice straw in the nylon bag occurred (31.5 % dry mass reduction, Additional file 1: Table S1). Further analysis showed that the mass reduction was largely attributed to the degradation of cellulose and hemicellulose, both of which accounted for 78.9 % of the decrease in dry mass during the enrichment phase. The remaining reduction was likely due to the degradation of other components of the rice straw, such as pectin, lignin, and protein. In addition, the effectiveness of the microbial enrichment was preliminarily verified by our observation of a high abundance of cellulolytic microbes from the post-enrichment rice straw on Congo red agar, while only very few microbes were found from the pre-enrichment rice straw (Additional file 2: Fig. S1). Together, these results demonstrate that the rice straw-adapted microbial consortia enriched from the compost ecosystem were capable of degrading lignocellulosic biomass and support previous observations that compost habitats harbor a large number of cellulolytic microbes [3, 7].

### Microbial diversity in RSA consortia enriched from manure compost

**Fig. 1:**
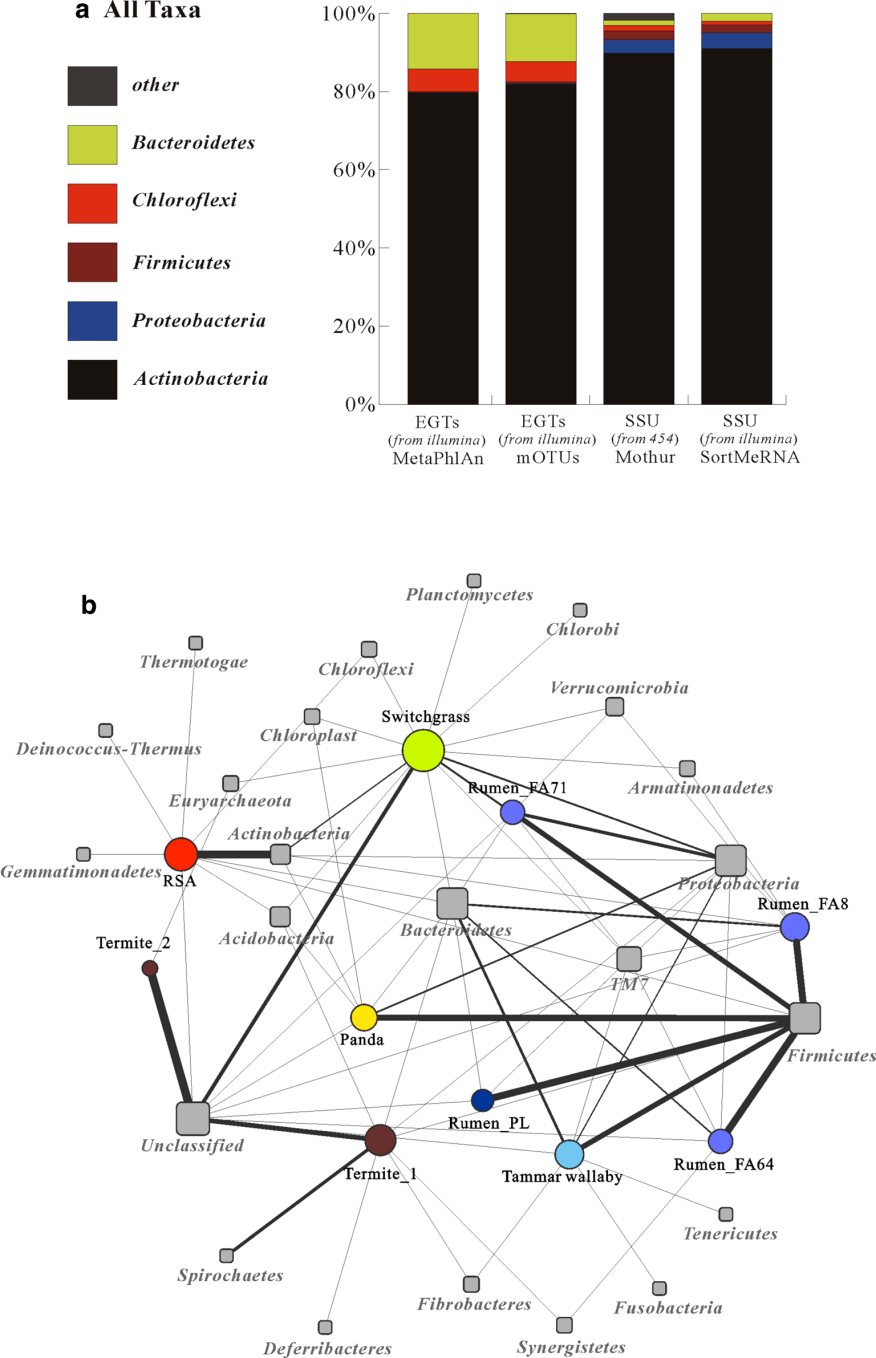
Phylogenetic composition of bacterial phyla from environmental gene tags (*EGTs*) and pyrosequence 16S rDNA sequences from the RSA consortia (**a**), and network for metagenome taxonomic profiling from the bovine, termite, panda, tammar wallaby samples as well as the RSA consortia (**b)**

Metagenomic sequencing of the RSA consortia yielded total read counts of 59,627,950 after quality filtering. We performed *de novo* assembly and predicted 174,022 open reading frames (ORFs). The average ORF length was 657 bp, and 39.7 % of the ORFs were predicted to represent full-length genes (Additional file 3: Table S2). To extend the taxonomic analysis, a 16S rRNA amplicon library was additionally generated. A total of 27,644 fragments spanning the V1–V3 variable region were analyzed. Both metagenomic and 16S rRNA amplicon datasets showed that the RSA consortia were primarily comprised bacterial members (>98 %), along with very few archaea and eukarya. Various analysis approaches were then used to accurately estimate the microbial diversity within the RSA consortia, such as MetaPhlAn, mOTUs, Mothur, and SortMeRNA [22–25]. While these analyses showed minor differences in the rank abundance order, the phylum Actinobacteria was the predominant group among the bacteria in the RSA consortia, followed by Proteobacteria, Firmicutes, Chloroflexi, and Bacteroidetes (Fig. 1a). To gain further insight into the diversity within the RSA consortia, the metagenomic dataset was taxonomically profiled using MetaPhlAn [22]. This tool can accurately identify specific microbial clades at the species level or higher taxonomic levels, thereby providing new insights into the complexity of microbial communities. The MetaPhlAn analysis showed that *Thermobispora bispora*, *Rhodothermus marinus*, *Sphaerobacter thermophilus* and *Thermomonospora curvata*, were extremely abundant, while the thermophile *Symbiobacterium*, *Thermobifida*, and *Geobacillus* species were presented at low abundance in the RSA consortia (Additional file 4: Table S3). Most of the species identified as being abundant in our samples have been detected previously in the environment of hot composts. It was reported that they are proficient degraders of plant biomass, especially the Actinobacteria, including *T. bispora,T. curvata*, and *Thermobifida fusca* [26–28]. The predominance of these thermophile species in the RSA consortia may be closely related to the increased biomass degradation in the high-temperature composting phase as has been reported in previous research [5, 29]. It should be also noted that the large proportion of lignocellulolytic species suggests that the enrichment technique we used could contribute to the achievement of the desired property in the RSA consortia.

Obviously, taxonomic profiling distinguished our metagenome from other typical and distinctive lignocellulose-degrading ecosystem metagenomes, which were derived from bovine, termite, panda, and tammar wallaby samples (Fig. 1b). The latter were dominated by the representatives of the Firmicutes and Bacteroidetes [10, 12, 30]. This incisive taxonomic disparity was most likely due to different environmental properties. For example, unlike the other lignocellulose-degrading ecosystems listed above, the compost ecosystem is characterized by high temperature, weak alkaline and aerobic conditions. These could significantly facilitate the growth of actinomycetes, which have been shown to exhibit a high plant biomass-degrading capacity [28, 31–33]. It is also worth noting that the taxonomic profile of our RSA consortia bore the greatest resemblance with that of the switchgrass-adapted compost community: both contained a number of actinomycetal members (Fig. 1b). However, presumably due to the differences in composting conditions and feedstock composition (i.e., rice straw vs. switchgrass), these two consortia substantially differed in the composition of the microbial community at the genus level. The actinobacterial genus *Stackebrandtia* was dominant in the switchgrass-adapted compost community [5], whereas *Thermobispora* was extremely abundant in our RSA consortia (Additional file 4: Table S3). Although both actinobacterial genera were reported to effectively degrade cellulose and hemicellulose [34, 35], their physiological adaptability and growth performance were considerably different. One of the largest discrepancies is their ability to grow at various temperatures. It was reported that the growth temperature range for *Stackebrandtia nassauensis* and *T. bispora* was 15 ~ 37 and 50 ~ 65 °C, respectively [36, 37]. These figures explain why thermophilic actinomycetes thrive in the RSA consortia but are scarcely detected in bovine, termite, panda, tammar wallaby, as well as switchgrass-adapted compost microbiomes. Based on the unique taxonomic profile of the RSA consortia, we hypothesize that these thermophilic communities may display distinctive functional properties and subsequently may be uniquely adapted to the lignocellulosic materials in the compost habitat.

### Microbial gene functions in RSA consortia enriched from manure compost

**Fig. 2:**
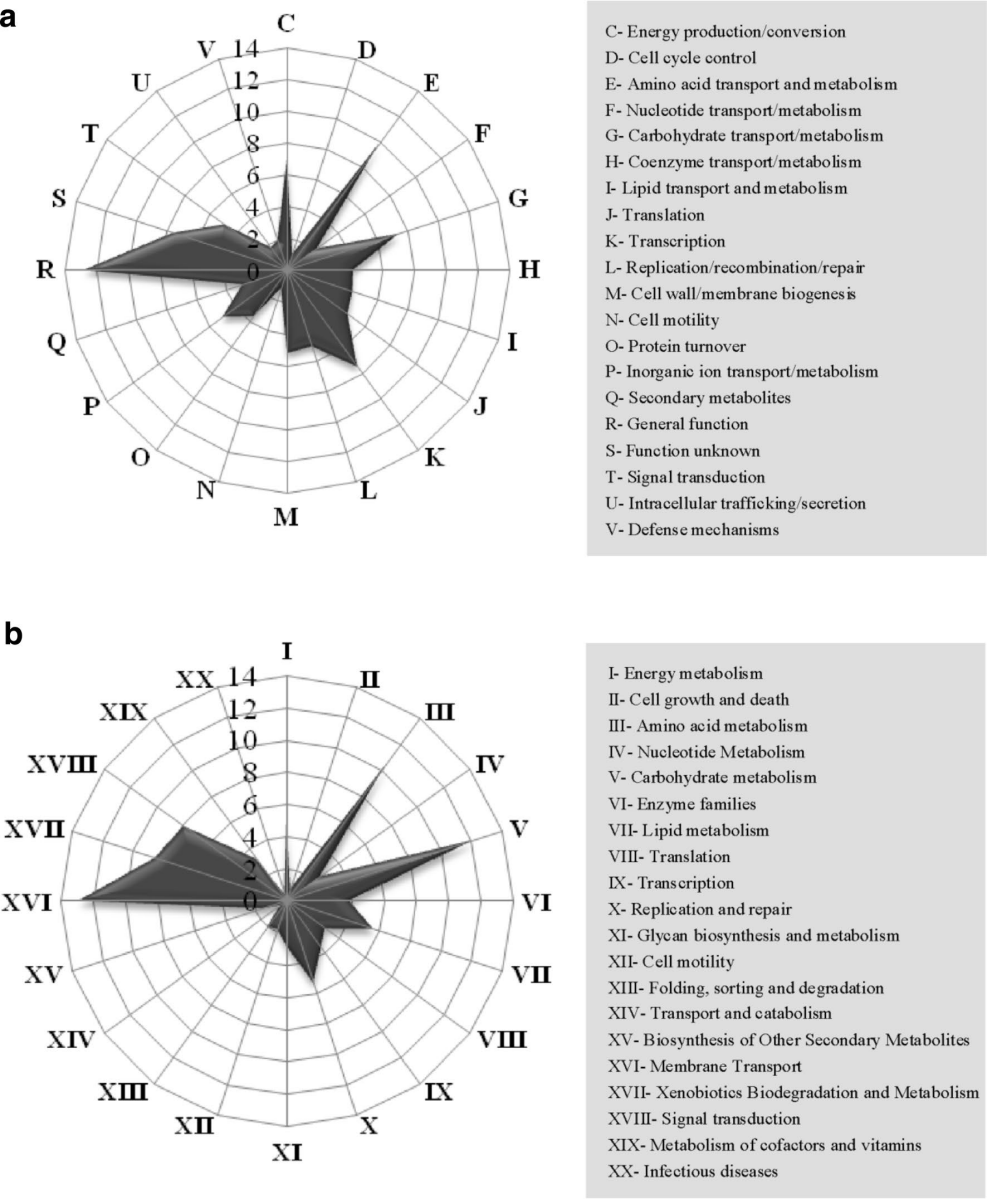
Distribution pattern of **a** COG-assigned and **b** KEGG-assigned proteins in the RSA consortia. Genes not assignable to any COGs or KEGGs are not shown in this figure. The percentage of matched gene numbers was assigned to specific COG or KEGG functional categories

Given that metagenomics can provide an initial picture of the physiological properties of microbial consortia, we compared the catalog with the COG and KEGG databases to assess the functions prominent in the RSA consortia. BLAST comparisons of all sequences yielded 124,378 hits for the COG database and 48,204 hits for the KEGG pathways. The enrichment of COG categories in Fig. 2a shows that the RSA consortia were enriched for general function (12.7 % in all COG functional categories), amino acid transport and metabolism (9.8 %), transcription (7.5 %), and carbohydrate transport/metabolism (7.3 %). The ontology analysis based on KEGG revealed similar patterns as shown in Fig. 2b, where membrane transport (12.7 % in all KEGG pathways), carbohydrate metabolism (12.1 %), amino acid metabolism (10.4 %), as well as xenobiotics biodegradation and metabolism (8.5 %) were abundant in the RSA consortia. Further, the comparative COG functional analysis of the RSA consortia with eight other well-known lignocelluloses-degrading microbiomes from termite, panda, 55 and 40 degree reactor, switchgrass-adapted compost, honey bee, wallaby and bovine rumen proved that they share similar metabolic patterns, particularly associated with transcription, carbohydrates and amino acids metabolism (Additional file 5: Fig. S2). These metabolic reconstructions suggest that the RSA consortia have enriched several functional capacities, especially, as expected, for carbohydrate metabolism. In order to determine the potential roles of microbial communities in the decomposition of plant polymer, specific COGs involved in carbohydrate transport and metabolism were analyzed in more detail (Additional file 6: Table S4). Notably, the RSA consortia harbored a diversity of genes involved in the metabolisms of the glucose, mannose, arabinose, galactose, glucuronose, xylose, and fructose, all of which accounted for 11.8 % of the COG subcategory G (Carbohydrate transport and metabolism). This suggests that the plant polysaccharides which the compost microbiome consumed were rich in glucan-, xylan-, pectin-, arabinose-containing carbohydrate structures, potentially supporting our previously mentioned observation that the lignocellulosic biomass reduction during the composting phase was mainly attributed to the degradation of cellulose and hemicellulose. Furthermore, the genes related to sugar/polysaccharide transport and phosphotransferase systems in the RSA consortia were more abundant than those encoding for polysaccharide-degrading enzymes (Additional file 6: Table S4). For example, the ATP-binding cassette (ABC) -type sugar transporters (COG1653, COG1175, COG0395, and COG1129), which may be involved in the uptake of carbohydrate [38], accounted for 17.7 % of the COG subcategory G. Another carbohydrate-related function enriched in the RSA consortia was the family of arabinose efflux permease (COG2814, 6.8 % of the COG subcategory G), which is responsible for the export and import of compounds such as sugars, antimicrobials, and amino acids [39]. Again, predicted genes related to phosphotransferase system (COG1925, COG1762, and COG1263) were detected. They might function in the transport and phosphorylation of numerous monosaccharides, disaccharides, amino sugars, polyols, and other sugar derivatives [40]. Altogether, the wide diversity of gene functions in carbohydrate metabolizing and transporting reveals a high potential of the RSA consortia for glycan degradation and sugar uptake and transport in the compost ecosystem.

### The abundance, variety and phylogenetic distribution of CAZymes in the RSA consortia

To better understand the carbohydrate metabolism in the compost ecosystem, where microorganisms synergistically degrade and utilize the cell wall components, we screened our metagenome for genes encoding enzymes that catalyze the hydrolysis of plant polymers. The RSA consortia contained 4810 different CAZyme genes distributed unequally between glycoside hydrolases (GHs, 35 %), carbohydrate-binding modules (CBMs, 13 %), polysaccharide lyases (PLs, 3 %), carbohydrate esterases (CEs, 18 %), and glycosyltransferases (GTs, 31 %). It is noteworthy that the CAZyme profile in the RSA consortia was close to the transcriptional profile of CAZyme genes in a maturing compost sample (unpublished data) (Additional file 7: Fig. S3). This finding suggests that carbohydrate metabolism pathways performed by CAZymes in the RSA consortia may to a large extent be expressed and active within the compost ecosystem at the time when we sampled.

**Fig. 3:**
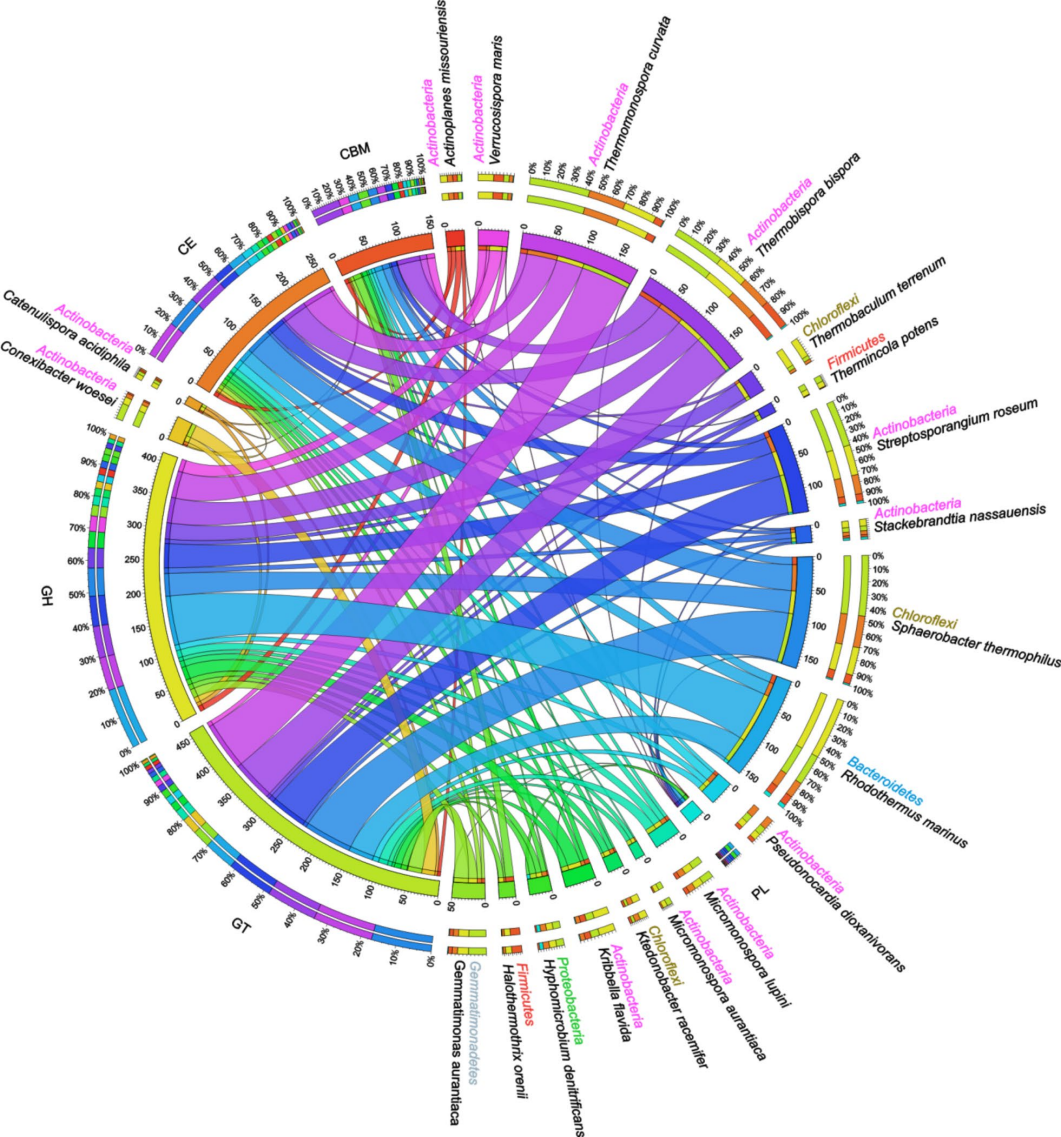
Phylogenetic distributions of carbohydrate-active enzymes in the most abundant members possessing CAZyme genes. The data were visualized via Circos software [84]. The width of *bars* from each microbial species and functional enzyme family indicates their relative abundance in the RSA consortia

Next, the overall phylogenetic distribution of CAZyme genes in the RSA consortia was analyzed to link compost microbial community composition and lignocellulose decomposition processes. The abundance of CAZyme genes varied across bacterial phyla. The majority of these genes in the RSA consortia were found from Actinobacteria (46.1 %), followed by Proteobacteria (16.7 %), Firmicutes (14.2 %), Chloroflexi (7.7 %), and Bacteroidetes (6.1 %), and more importantly, the members from Actinobacteria were dominant in all carbohydrate-active modules (GHs, GTs, PLs, and CEs) and their associated CBMs (Additional file 8: Fig. S4). Since the content of CAZyme genes was not homogeneously distributed within each phylum, we also investigated the detailed phylogenetic distribution of genes involved in lignocelluloses degradation at lower taxonomic levels. Intriguingly, twelve different species of phylum Actinobacteria were present in the 20 most abundant members possessing CAZyme genes, revealing that the phylum Actinobacteria harbored much more CAZyme genes and exhibited a much wider distribution of CAZyme repertoire in the RSA consortia (Fig. 3). These findings, taken together with the fact that the phylogenetic distributions of CAZyme genes largely distinguished the RSA consortia (predominantly Actinobacteria) from that of the bovine rumen (*Clostridiales*), the termite hindgut (Fibrobacteres and Spirochetes), the honey bee gut (Proteobacteria), and the Tammar wallaby foregut (Bacteroidetes) [10, 11, 13, 30], strongly emphasized the functional versatility and distinctive adaptation of actinobacterial communities in the processing and metabolizing of carbohydrates within the compost habitat. However, in addition to *T. bispora* (8.79 %), *T. curvata* (8.56 %), and *Streptosporangium roseum* (6.43 %) of the phylum Actinobacteria, the CAZyme genes were also mainly detected in *S. thermophilus* (7.99 %) of the phylum Chloroflexi as well as *R. marinus* (7.47 %) of the phylum Bacteroidetes (Fig. 3). There are several implications of this observation: Firstly, the composition of functional members corresponds well to the structure of the ecologically dominant species in the RSA consortia. Potentially, a link exists between microbial taxa and their functional traits. This suggests that polysaccharides within the compost habitat are hydrolyzed by the predominant Actinobacteria in cooperation with Chloroflexi and Bacteroidetes. Further, although high levels of cellulose-degrading enzymes were found previously to be secreted by *T. bispora*, *T. curvata*, and *R. marinus* [41–43], our data provide genetic evidence for both highly cellulolytic and highly transglycosylatic abilities of these well-characterized strains, which harbor an abundant of genes encoding GHs and GTs.

As a widespread group of enzymes that hydrolyze the glycosidic bond between carbohydrates, GH families were most attached to oligosaccharide-degrading enzymes (18.18 %), along with relatively few to cellulases (4.60 %) and endohemicellulases (4.90 %) (Table 1). This vast majority of oligosaccharide-degrading enzymes identified in our metagenome suggests that the RSA consortia were predominantly processing and metabolizing the partially degraded plant materials in the compost ecosystem. In particular, β-glucosidases responsible for the processing of small oligosaccharides in the RSA consortia were mostly present in GH3 and GH1 (8.62 %), whereas the endo- and exo-cellulases were predominantly found in GH5 and GH9 (3.48 %) (Table 1). A range of CAZymes targeting hemicelluloses (such as GH10 xylanases and GH26 mannanase, 3.01 %) and pectic polysaccharides (PL9 pectate lyase) were also abundant (Additional file 9: Table S5). Together with the COG profiles for glycan degradation, the diverse repertoire of CAZymes systems provides the basis for a collaborative system efficient in the processing and metabolizing of carbohydrates in the compost habitat. However, among the total of 85 GHs, the largest two sequence-based families were the families of GH109 and GH13 (Additional file 9: Table S5), known as the dehydrogenase and α-amylase family associated with starch biosynthesis and turnover [44]. The next most abundant is the GH23 family, among which nearly half were derived from Actinobacteria. This family was thought to possess inverting lysozymes and lytic transglycosylases that cleave glycosidic bonds via a water-independent substrate-assisted mechanism [45]. In addition to these putative GHs, we also detected a number of CBM2 and CBM50, in which Actinobacteria accounted for 95.5 and 9.8 %, respectively. These non-catalytic CBMs are contiguous amino acids within the carbohydrate-active enzyme with a discreet fold promoting efficient adherence of the enzyme to insoluble substrates and thus enhancing the catalytic cleavage of the substrate [46–48]. It is plausible that the abundance of GH23 together with CBM2 and CBM50, leads to the efficiency of the dominant populations in the RSA consortia, especially of the Actinobacteria, to degrade the insoluble lignocellulosic plant biomass in the solid condition of the compost habitat.

**Table 1:** Glycoside hydrolase profiles targeting plant structural polysaccharides in four metagenomes

	Predominant activity	Termite [11]	Bovine [10]	Macropod [30]	RSA
Cellulases					
GH5	Cellulases	7.97	1.23	4.31	2.36
GH6	Endoglucanases	0.00	0.00	0.18	0.77
GH7	Endoglucanases	0.00	0.00	0.00	0.00
GH9	Endoglucanases	1.28	0.92	0.36	1.12
GH44	Endoglucanases	0.85	0.00	1.08	0.06
GH45	Endoglucanases	0.57	0.00	0.00	0.00
GH48	Cellobiohydrolases	0.00	0.00	0.00	0.30
Subtotal (%)		10.67	2.15	5.92	4.60
Endohemicellulases					
GH8	Endoxylanases	0.71	0.61	0.36	0.00
GH10	Endo-1,4-β-xylanases	6.54	1.08	2.51	2.18
GH11	Xylanases	1.99	0.15	0.00	0.47
GH12	Xyloglucanases	0.00	0.00	0.00	0.41
GH26	β-mannanase & xylanases	2.13	0.77	2.33	0.83
GH28	Galacturonases	0.85	0.77	0.54	0.65
GH53	Endo-1,4-β-galactanases	1.71	2.61	1.62	0.35
Subtotal (%)		13.94	5.99	7.36	4.90
Debranching enzymes					
GH51	α-L-arabinofuranosidases	2.56	9.83	2.33	1.42
GH54	α-L-arabinofuranosidases	0.00	0.15	0.00	0.00
GH62	α-L-arabinofuranosidases	0.00	0.00	0.00	0.35
GH67	α-glucuronidases	1.42	0.00	0.90	0.35
GH78	α-L-rhamnosidases	0.00	5.22	4.49	2.30
Subtotal (%)		3.98	15.21	7.72	4.43
Oligosaccharide-degrading enzymes					
GH1	β-glucosidases	3.13	1.54	11.13	3.72
GH2	β-galactosidases	3.27	28.57	5.03	1.59
GH3	β-glucosidases	9.82	27.04	13.46	4.90
GH29	α-L-fucosidases	0.00	11.37	0.54	0.89
GH35	β-galactosidases	0.43	1.84	0.72	0.41
GH38	α-mannosidases	1.56	2.61	0.54	0.77
GH39	β-xylosidases	0.43	0.31	0.18	1.89
GH42	β-galactosidases	3.41	1.69	1.62	0.83
GH43	Arabino/xylosidases	2.28	9.37	3.41	3.01
GH52	β-xylosidases	0.43	0.00	0.00	0.18
Subtotal (%)		24.75	84.33	36.62	18.18
Total GHs		703	651	557	1694
% ORFS		0.78	0.78	0.71	0.97

The listed value is the population abundance weighted relative abundance (%) of GH families among the total GHs

Although most of these CAZymes profiles of the RSA consortia were similar to those of the termite, bovine and macropod microbiomes, 0.97 % of the sequences assigned to the GH families in the RSA consortia are subtly greater than those in the termite (0.78 %), bovine (0.78 %), and macropod (0.71 %) communities (Table 1). Considering the fact that the numbers of hydrolytic enzymes produced by the anaerobic gut habitats are relatively lower than those observed for their aerobic counterparts [49], the higher percentage of average GH families in the aerobic compost habitat indicates a much more abundant and diverse set of microbial genes directed towards accessing a wide range of polysaccharides. Furthermore, compared to these herbivore microbiomes, the RSA consortia contained a certain amount of genes associated with cellulosome complexes (e.g., GH48, 0.30 %), which facilitate interactions between the insoluble substrate and the bacterial enzymatic machinery [50]. Other differences in the GHs profile of these four metagenomes include the presence of the GH12 (0.41 %) and GH62 (0.35 %) families as well as the absence of the GH8 family in our study. It is important to note that GH12 and GH62 mainly originated from Actinobacteria (75 and 100 %, respectively). The detection of GH12, a thermostable endoglucanase with a high efficiency for rice straw saccharification [51, 52], further confirms the high capacity of Actinobacteria for degrading rice straw in the thermophilic composting environment. Again, the existence of GH62 supports that Actinobacteria possess several accessory enzymes, which could facilitate the arabinoxylan degradation [53]. The diverse repertoire and distinct phylogenetic distribution of CAZymes in the RSA consortia, therefore, further reflect these typical cellulolytic characteristics (thermostability and versatility) of actinomycetal community along with its capacity of attacking the insoluble substrates, hence contributing to the unique adaptation of composts-specific microbial populations to lignocellulosic plant biomass.

### A systematic overview of lignocellulose degradation by the RSA consortia

Lignocellulose of plant biomass is chemically complex consisting of some major polymers, such as cellulose, hemicellulose, pectin, and lignin. In nature, the efficient degradation of lignocellulosic biomass involves a cooperative action of various microorganisms producing multiple enzymes that act specifically and synergistically in the degradation of plant biomass. Therefore, in this study, the cultivation-independent metagenomic approach was applied to provide an overview of microbial degradation of primary plant cell wall polysaccharides along with lignin by individual members of the RSA consortia, which were effectively enriched from the compost habitat. Specifically, we examined the carbohydrate-digestive capacity of the RSA consortia, determined the top Blast-hit organism of each identified enzyme at the species level, and interpreted the ecosystem functions of major microorganisms and their potential synergistic action in these complex processes.

### Cellulose bioconversion

**Fig. 4:**
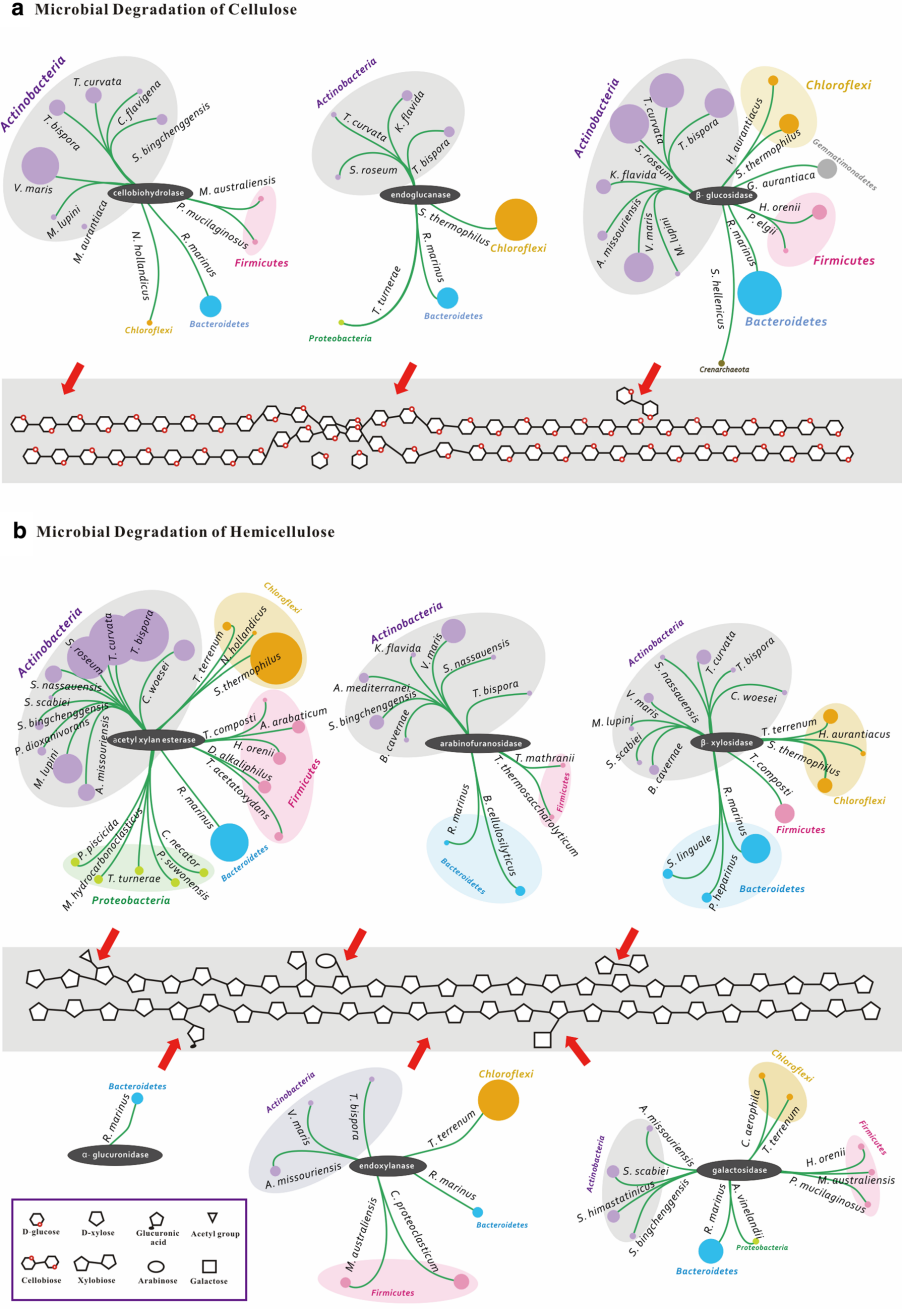
An overview of microbial degradation of cellulose and hemicellulose in the RSA consortia. We show the dominant species that digest the cellulose and hemicellulose by depicting the distribution of genes encoding the CAZymes in the RSA consortia. The *purple*, *pink*, *green*, *blue*, *yellow*, *gray* and *brown* *circles* represent the members of phylum Actinobacteria, Firmicutes, Proteobacteria, Bacteroidetes, Chloroflexi, Gemmatimonadetes and Crenarchaeota, respectively. The diameter of each circle is proportional to its relative abundance. *A. arabaticum, Acetohalobium arabaticum*; *A. mediterranei, Amycolatopsis mediterranei*; *A. missouriensis, Actinoplanes missouriensis*; *A. vinelandii, Azotobacter vinelandii*; *B. cellulosilyticus, Bacteroides cellulosilyticus*; *B. cavernae, Beutenbergia cavernae*; *C. aerophila, Caldilinea aerophila*; *C. proteoclasticum, Clostridium proteoclasticum*; *C. flavigena, Cellulomonas flavigena*; *C. necator, Cupriavidus necator*; *C. woesei, Conexibacter woesei*; *D. alkaliphilus, Dethiobacter alkaliphilus*; *G. aurantiaca, Gemmatimonas aurantiaca*; *H. aurantiacus, Herpetosiphon aurantiacus*; *H. orenii, Halothermothrix orenii*; *K. flavida, Kribbella flavida*; *M. aurantiaca, Micromonospora aurantiaca*; *M. australiensis, Mahella australiensis*; *M. hydrocarbonoclasticus, Marinobacter hydrocarbonoclasticus*; *M. lupini, Micromonospora lupini*; *N. hollandicus, Nitrolancetus hollandicus*; *P. heparinus, Pedobacter heparinus*; *P. piscicida, Pseudoalteromonas piscicida*; *P. dioxanivorans, Pseudonocardia dioxanivorans*; *P. elgii, Paenibacillu elgii*; *P. maris, Planctomyces maris*; *P. mucilaginosus, Paenibacillus mucilaginosus*; *P. suwonensis, Pseudoxanthomonas suwonensis*; *R. marinus, Rhodothermus marinus*; *S. bingchenggensis, Streptomyces bingchenggensis*; *S. clavuligerus, Streptomyces clavuligerus*; *S. hellenicus, Staphylothermus hellenicus*; *S. himastatinicus, Streptomyces himastatinicus*; *S. linguale, Spirosoma linguale*; *S. nassauensis, Stackebrandtia nassauensis*; *S. roseum, Streptosporangium roseum*; *S. scabiei, Streptomyces scabiei*; *S. thermophilus, Sphaerobacter thermophilus*; *T. acetatoxydans, Tepidanaerobacter acetatoxydans*; *T. curvata, Thermomonospora curvata*; *T. terrenum, Thermobaculum terrenum*; *T. turnerae, Teredinibacter turnerae*; *T. bispora, Thermobispora bispora*; *T. composti, Thermobacillus composti*; *T. mathranii, Thermoanaerobacter mathranii*; *T. potens, Thermincola potens*; *T. thermosaccharolyticum, Thermoanaerobacterium thermosaccharolyticum*; *V. maris, Verrucosispora maris.* The *bubble plot* indicates the relative abundances of each microbial species in the RSA consortia

The classical scheme for cellulose degradation involves at least three different classes of hydrolytic enzymes to participate: endoglucanases for catalyzing random cleavage of internal bonds in the cellulose chain, cellobiohydrolases for attacking the chain ends and hence releasing cellobiose, and β-glucosidases for acting on cello-oligosaccharides and cellobiose and subsequently releasing glucose. Genes encoding for these putative cellulases in the RSA consortia were mainly from the families GH1, GH3, GH5, GH6, GH9, GH12, GH44, GH48, and GH74 (Additional file 9: Table S5). Clearly, cellulolytic species in the RSA consortia were remarkably diverse (Fig. 4a). Endoglucanase was predominantly present in thermotolerant *S. thermophilus* and *R. marinus*, while both cellobiohydrolase and β-glucosidase activities were mainly derived from members of the Actinobacteria, such as *Verrucosispora maris*, *T. curvata,* and *T. bispora*. This strongly suggests a synergistic action of multiple members affiliated to Actinobacteria, Chloroflexi, and Bacteroidetes during the degradation of cellulose. The synergistic pattern shown in the compost habitat differs substantially from the rumen microbiome, in which cellulolytic activities were performed by Firmicutes, Fibrobacteres and Bacteroidetes [50] and from the termite hindgut microbiome, in which these processes were dominated by Spirochaetes and Fibrobacteres [11].

It has been generally accepted that the β-glucosidase activity is a key factor in the cellulolytic process [54]. In this study, the putative β-glucosidase was the most abundant enzyme. Its phylogenetic distribution was the most diverse among the three classes of hydrolytic enzymes, mainly from a variety of Actinobacteria specie including *V. maris*, *T. curvata*, *T. bispora*, *S. roseum*, and *Kribbella flavida*. Of these cellulolytic species, *S. roseum* and *K. flavida* were firstly detected to participate in the degradation of cellulose in the compost habitat, although *V. maris*, *T. curvata* and *T. bispora* have been frequently identified as primary actinomycetes for cellulose degradation in natural habitats [55]. More importantly, the strictly thermophilic *T. bispora*, as the predominant member in the RSA consortia, has the potential to carry out the functions of three classes of cellulolytic enzymes as well as those of some xylan-degrading enzymes (Fig. 4a, b). This suggests a greater phylogenetic and functional diversity of actinomycetal communities involved in cellulose degradation than previously assumed.

### Hemicellulose bioconversion

During the enzymatic hydrolysis of hemicellulose, endoxylanase, β-xylosidase, α**-**glucuronidase, arabinofuranosidase, galactosidase, and acetylxylan esterase all act on the different heteropolymers available in lignocellulosic biomass (Fig. 4b). Genes encoding for these putative hemicellulases in the RSA consortia were mainly from the families GH1–GH4, GH10, GH11, GH27, GH35, GH36, GH39, GH43, GH51, GH52, GH62, GH67, GH127, CE1, CE3, CE4, and CE7 (Additional file 9: Table S5). Strikingly, the dominant members associated with hemicellulose bioconversion in the RSA consortia were also thermophilic. *Thermobaculum terrenum* of the phylum Chloroflexi was predominantly responsible for the hydrolysis of the internal glycosidic linkages of the heteroxylan backbone (endoxylanase), whereas *Rhodothermus marinus* of the phylum Bacteroidetes was mainly responsible for degrading short molecules such as xylooligosaccharide, xylobiose, galactolipid, and glucuronide. These findings indicate that the members mainly from Bacteroidetes along with Chloroflexi probably had a crucial role in the degradation of hemicellulose, specializing towards its glycosidic linkage hydrolysis under the thermophilic conditions of the compost habitat. It should also be noted that the key role of Bacteroidetes in degrading hemicellulose polymers was confirmed by a previous report on the metagenome of the mesophilic fermenting platform community [15]. However, unlike this community, the dominant species of the phylum Bacteroidetes functioning in the RSA consortia is *R. marinus*, which is an extreme thermophilic bacterium thriving in various thermal environments and displaying diverse inherent enzyme activities in degrading lignocellulosic biomass [56].

Of note, however, is that the actinomycetal population containing genes for encoding endoxylanase and β-xylosidase was less well represented but more diverse, including the following species *T. bispora*, *T. curvata*, *S. roseum*, *Micromonospora lupini*, *V. maris*, *Conexibacter woesei*, *Stackebrandtia nassauensis*, *Beutenbergia cavernae*, *Streptomyces scabiei*, *and Actinoplanes missouriensis*. Most of these actinomycetal species were reported to be able to simultaneously produce a number of acetyl xylan esterases and arabinofuranosidases, which hydrolyze the acetyl substitutions on xylose moieties and arabinofuranosyl linkages in arabinoxylan, respectively [57]. Considering the synergisms between esterases/arabinofuranosidases and xylanases in the enzymatic degradation of xylan [57, 58], we suggest that the prominent actions of actinomycetal esterase and arabinofuranosidase may greatly facilitate the efficient and complete the breakdown of xylan during the bioconversion of hemicelluloses.

### Pectin bioconversion

**Fig. 5:**
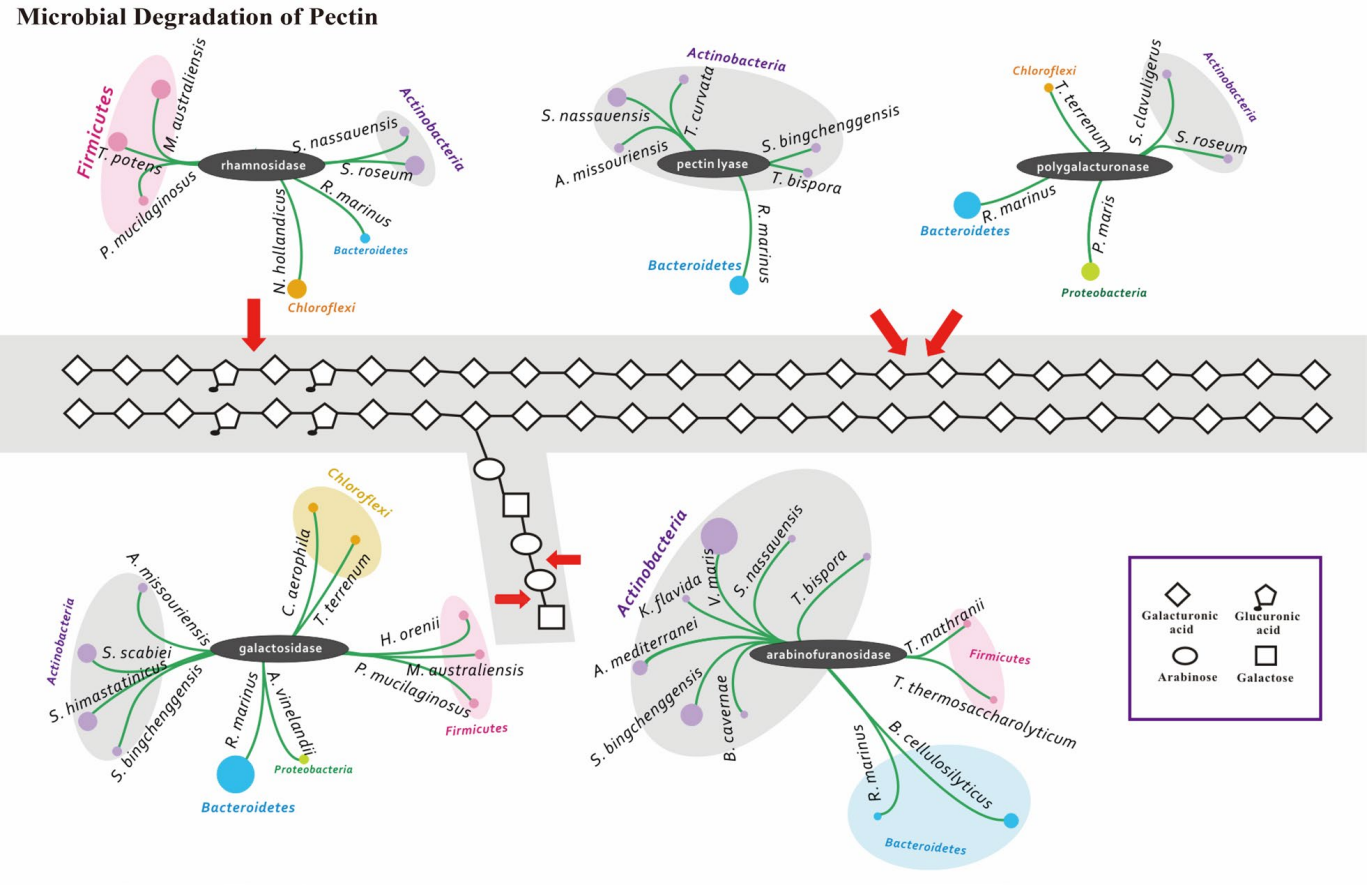
As in Fig. 4 but for microbial degradation of pectin in the RSA consortia

Pectin, a family of covalently linked galacturonic acid-rich polysaccharides, is the most structurally and functionally complex polysaccharide of plant cell walls [59]. Complete hydrolysis of pectins involves a set of diverse enzymes (Fig. 5), including pectin lyase, polygalacturonase, galactosidase, arabinofuranosidase, and rhamnosidase. Genes encoding for these putative pectinases in the RSA consortia were mainly from the families PL1, PL3, PL4, PL9, PL10, GH2, GH4, GH27, GH28, GH35, GH36, GH51, GH62, GH78, GH106, and GH127 (Additional file 9: Table S5). Among these known pectinases, pectin lyase has an essential role in the pectin bioconversion since it is the only enzyme that can cleave the α-1, 4 bonds of highly esterified pectins without prior actions of other enzymes. It has been generally accepted that the microbial sources of pectin lyase are specific fungi and bacteria, such as *Aspergillus niger*, *Bacillus* spp., and *Pseudomonas* spp. [60–63]. However, in our study, a range of rare actinomycetes was the major producer of this important enzyme, especially *Stackebrandtia nassauensis*, *Actinoplanes missouriensis*, *T. bispora*, and *T. curvata*. While pectin lyase has been previously detected in *Streptomyces viridochromogenes* and *Thermomonospora flisca* [64, 65], so far very limited information is available on the pectinolytic enzyme system of the class Actinobacteria. This first report on pectin lyase production by rare actinomycetes highlights their importance in the degradation of pectin in the compost ecosystem.

### Lignin bioconversion

As the main non-carbohydrate structural component of the plant cell wall, lignin is a highly complex aromatic heterogeneous polymer that is covalently cross-linked with polysaccharide fibers by covalent bonds [66]. Lignin attack is an oxidative process and requires a suite of oxidative enzymes, such as lignin peroxidase (LiP), manganese peroxidase (MnP), versatile peroxidase (VP), and laccase [67]. Genes encoding for these putative ligninases were from the family AA2 in the “Auxiliary Activities” (AAs) groups, and they accounted for 8.7 % of all AAs (Additional file 10: Table S6). After examining the phylogenetic distribution of genes in the family AA2, we found that in addition to *Symbiobacterium thermophilum* of the phylum Firmicutes, the dominant members involved in the degradation of lignin compounds in the RSA consortia were mainly from a variety of Actinobacteria species, including *T. curvata*, *Mycobacterium xenopi*, *Amycolicicoccus subflavus,* and *Mycobacterium thermoresistibile*. The diversity of these previously unrecognized members greatly increases the inventory of ligninases since actinomycetes identified by in vitro assays for lignin degradation were affiliated within a limited number of genera, including *Streptomyces*, *Rhodococcus* and *Nocardia* [68].

### Implications

In recent years, thermostable lignocellulases have been a target for metabolic engineering related to biomass conversion and biofuel production [69]. The compost habitat, characterized by high temperature (~70 °C) and abundant lignocellulosic materials (e.g., rice straw, dry leaves, sawdust), is potentially a site for prospecting thermophilic biomass-degradation enzymes [70]. As expected, our pyrosequencing and metagenomic analyses of the RSA consortia revealed that the numerically dominant and deeply branched thermophilic members from Actinobacteria have the potential to effectively degrade the lignocellulosic biomass in the compost habitat. Properties of the GHs and CBMs found in this study indicated that lignocellulosic enzymes produced by Actinobacteria in composts might be thermostable, which is supported by the previous finding that cellulase derived from *Streptomyces* sp. was active at 37 ~ 75 °C [71]. Therefore, from an applied point of view, the compost-specific lignocellulases operating stable under a range of high temperatures would have advantages in industrial lignocellulose processing compared to the most commonly used commercial enzyme cocktails produced by the fungus *Trichoderma reesei* with an optimum temperature range between 40 and 50 °C [72].

To our knowledge, the present study is the first research illustrating a synergistic action of the predominant Actinobacteria along with Chloroflexi and Bacteroidetes and Firmicutes in the hydrolysis of polysaccharides within the compost habitat. It is also the first study that provides genetic evidence supporting that compost microbiome contained a variety of previously known and so far unrecognized actinomycetal members potentially displaying versatile lignocelluloses-degrading abilities, especially in the β-glucosidase, acetyl xylan esterase, arabinofuranosidase, pectin lyase and ligninase activity. These data reveal a greater ecological functional diversity of thermophilic Actinobacteria in carbohydrate metabolism in the compost habitat than previously assumed, underlining that there is still a broad swath of actinomycetes to be explored as potential candidates for mining efficient lignocellulosic enzymes in the biofuel industry.

It should be also noted that lignocellulose degradation within the compost habitat is a continuous process of dynamic interactions between complex microbial community and recalcitrant substances. It would be desirable to apply broad sampling and adopt a broad array of approaches, including metatranscriptomics, proteomics and metabolomics, so as to temporally identify and quantify the expression of genes and metabolic functions associated with the bioconversion of the major components of plant biomass during the composting period, such as cellulose, hemicellulose, pectin, lignin as well as silica, although pioneering our metagenomic study provided a first glimpse into the microbial enzymatic decomposition of lignocelluloses within the compost ecosystem.

## Conclusions

Pyrosequencing and metagenomic analyses revealed that the RSA consortia enriched from composting ecosystems contained a distinctive set of microorganisms, including Actinobacteria, Proteobacteria, Firmicutes, Chloroflexi, and Bacteroidetes. The diverse and distinct repertoire of CAZyme genes in the RSA consortia were also primarily distributed within these bacteria phyla, which suggests a synergistic system efficient in the processing and metabolizing of carbohydrates in the compost habitat. Interestingly, 46.1 % of the CAZyme genes were from Actinobacteria with an expanded catalog of cellobiohydrolase, β-glucosidase, acetyl xylan esterase, arabinofuranosidase, pectin lyase, and ligninase genes. The versatile functions illustrate the importance of compost actinomycetal communities in the bioconversion of lignocellulosic biomass. In addition, the detection of a variety of previously known and so far unrecognized actinomycetal members in the RSA consortia highlights the power of moving beyond surveys of microbial diversity and mining enzymes from microbial communities for biofuel industry by functional-driven metagenomics.

## Methods

### Enrichment of rice straw-adapted microbial consortia in a compost habitat

We chose rice (*Oryza sativa* L.) straw as the feedstock to be degraded since it is more resistant to microbial degradation than the straw from other protein-rich plants such as wheat [73]. Air-dried rice straw was ground to pass a 2-mm sieve using a Retsch ZM200 rotor mill. The ground material (14.81 g) was weighed into a nylon bag with a mesh size of 50 μm. This nylon bag was placed in the middle of an aerobic windrow compost pile of cow manure, which was set up in a suburb of Hangzhou city in China. Aeration of the compost pile occurred via natural ventilation and turning, and the pile was periodically remixed and turned over with a wheel loader. When the pile temperature (29.2 °C) dropped to ambient level towards the end of the maturing stage of composting (day 30), the nylon bag was retrieved from the compost. Rice straw samples of 10.15 g were immediately collected from the bag and shipped to the laboratory on dry ice. To determine the extent of degradation of this substrate incubated in compost ecosystems, the chemical composition (cellulose, hemicellulose and lignin) of pre- and post- enrichment rice straw was determined according to the method described by Liu [74].

### Nucleic acid extraction

High molecular weight DNA was extracted from the microbial consortia adherent to the post-enrichment rice straw samples using the MoBio UltraClean Soil DNA isolation kit (MoBio Laboratories, Solana Beach, CA, USA). The extraction was performed according to the manufacturer’s instructions. The extracted DNA was 225 ng/ml and had a 260/280 ratio of 1.91 (Nanodrop, Thermo Scientific, Wilmington, DE, USA). The DNA was eluted in a final volume of 100 μL and the eluents were stored at −20 °C until analysis.

### High-throughput 16S rRNA gene pyrosequencing and phylogenetic classification

An amplicon library was constructed for 454 pyrosequencing using bacterial primers 8F (5′-AGAGTTTGATCCTGGCTCAG-3′) and 533R (5′-TTACCGCGGCTGCTGGCAC-3′) for the V1–V3 region of the 16S rRNA gene. The length of the amplicon, including the barcode and 454 primers, was ~596 nt. The PCR reaction was performed in a 25 μL mixture containing 22.5 μL of Platinum PCR SuperMix (Invitrogen, Shanghai, China), 0.5 μL of each primer at 30 μmol L^−1^, and 1.0–1.5 μL template DNA. The amplification conditions were as follows: 94 °C for 5 min, 28 cycles of 94 °C for 45 s, 55 °C for 30 s, and 72 °C for 45 s followed by extension at 72 °C for 5 min. The PCR products were purified using a 2.0 % agarose gel and quantified using Picogreen (Invitrogen, Shanghai, China). Our sample was subjected to a single pyrosequence run by a Roche massively parallel 454 GS-FLX according to standard protocols [75]. To minimize the effects of random sequencing errors, low-quality sequences were removed by eliminating those without an exact match to the forward primer, without a recognizable reverse primer, length shorter than 200 nucleotides and contained any ambiguous base calls (Ns). After trimming the barcodes and primers from the resulting sequences, we obtained 21,670 high-quality V1–V3 tags of the 16S rRNA gene. Sequences were clustered into an operational taxonomic unit (OTU) at a 0.03 cut-off using the MOTHUR program. The taxonomy of the high-quality reads was further classified using the RDP classifier with a confidence threshold of 80 % and the RDP taxonomic nomenclature [76]. All sequences have been deposited in GenBank short-read archive SRR1292608.

### Metagenome processing: DNA library construction and sequencing

DNA library construction and sequencing were performed by BGI (Shenzhen, China) and followed BGI’s previous work on human gut microbe metagenomic sequencing [77]. A library with 180-bp clone insert size was constructed for our sample. Sequence reads were quality trimmed to an accuracy of 99.4 % and duplicate reads were identified and removed prior to assembly. Nearly 59.6 million high-quality reads were generated, and the total data volume of high-quality reads was approximately 5 Gbp (Additional file 3: Table S2).

### Illumina genome analyzer short reads *de novo* assembly

High-quality short reads of the DNA sample were assembled by the SOAPdenovo assembler [78], as used in human gut microbe metagenomic analyses. After assessing different Kmer sizes, we found that Kmer 41 provided the longest contig length and highest read utilization rate. Consequently, we used the contigs of Kmer 41 for our sample as the final assembly result (Additional file 11: Table S7).

### Taxonomic profiling and functional classification in metagenomic database

The RSA consortia metagenomic dataset was taxonomically profiled using MetaPhlAn and mOTU [22, 23]. In order to further confirm the composition of microbial community in the RSA consortia, rRNA sequences of the small subunit (SSU) were extracted from the Illumina data using SortMeRNA [25]. Annotations by the MG-RAST pipeline [79] showed that our metagenome protein coding sequences contained 0.4 % RNA genes and 99.6 % protein coding genes, of which 63.3 % have been assigned a putative function. We performed predicted gene functional classification by querying protein sequences of the genes against the evolutionary genealogy of genes: non-supervised orthologous groups (eggNOG) database and the Kyoto Encyclopedia of Genes and the Genomes (KEGG) database using BLASTP with E-value <1 × 10^−5^. We used the “function comparison” tool of integrated microbial genomes with microbiome samples (IMG/M, http://img.jgi.doe.gov/cgi-bin/m/main.cgi, a data-management and analysis platform for genomic and metagenomic data based on IMG [80]) to compare the clusters of orthologous groups (COG) category of the RSA consortia against eight other microbiomes available on IMG/M including termite, panda, 55 and 40 degree reactor, switchgrass-adapted compost, honey bee, wallaby, and bovine rumen [5, 10–13, 15, 30].

### Carbohydrate-active enzymes (CAZymes): annotation and phylogenetic analysis

Searches for CAZymes were performed as described in Liu et al. [81]. Briefly, protein coding genes identified in the RSA consortia metagenome were searched (E < 10^−4^) against the set of profile hidden markov models (HMMs) representing the signature domain of each CAZy family defined by Yin et al. [82], as deposited on dbCAN (http://csbl.bmb.uga.edu/dbCAN/). The phylogenetic origins of CAZymes-encoding genes were determined using MEGAN [83].

### Sequence data submission

Datasets are publicly available on the integrated microbial genomes with microbiome samples (IMG/M, project ID 12014) and the metagenomics RAST server (MG-RAST, ID 4513787.3).

## Additional files

10.1186/s13068-016-0440-2 Comparison of the content of pre- and post- enrichment rice straw and its components (34 k).
10.1186/s13068-016-0440-2 Cultures grown on Congo red agar plates from post-enrichment rice straw (left) and pre-enrichment rice straw (right). About 1 g sample was added into 100 mL of sterile water and shaken at 150 rpm for 1 h. Then 100 μL of the resultant solution was inoculated on Congo red agar plates at 28 °C for 7 days. The Congo red agar medium contained K_2_HPO_4_ (0.5 g), MgSO_4_ (0.25 g), CMC-Na (1.88 g), yeast extract (1.0 g), Congo-red (0.4 g), agar (14.0 g), gelatin (2.0 g), pH 7.0 (per liter) (89 k).
10.1186/s13068-016-0440-2 Summary of illumina GA reads and ORF prediction (33 k).
10.1186/s13068-016-0440-2 MetaPhlAn species abundances in the RSA consortia (41 k).
10.1186/s13068-016-0440-2 Metabolic clustering of RSA consortia, termite, panda, reactors, switchgrass-adapted compost, honey bee, wallaby, and bovine rumen metagenomes. The different color represents relative percentage of the metabolic classes within each sample (112 k).
10.1186/s13068-016-0440-2 Glycan metabolism COGs enriched in rice straw-adapted microbial consortia enriched from manure compost (89 k).
10.1186/s13068-016-0440-2 A heat map cluster representing CAZyme profiles in the RSA consortia Metagenome and four typical sample Metatranscriptomes (including samples from the mesophilic stage, thermophilic stage, cooling stage and maturing stage of the composting process; unpublished data). The relative abundances of gene and transcript in Metegenome and Metatranscriptome, respectively, were standardized by z-score transformation (236 k).
10.1186/s13068-016-0440-2 Phylogenetic distributions of carbohydrate-active enzymes at the phylum level in the RSA consortia. The data were visualized via Circos software (427 k).
10.1186/s13068-016-0440-2 Summary of carbohydrate-active enzymes found in the rice straw-adapted consortia enriched from manure compost (262 k).
10.1186/s13068-016-0440-2 Summary of Auxiliary Activities (AA) families found in the rice straw-adapted consortia enriched from manure compost (39 k).
10.1186/s13068-016-0440-2 Summary of *de novo* assembly results (37 k).

## Abbreviations

ABC: ATP-binding cassette
bp: base pairs
CAZy: carbohydrate-active enzymes
CBM: carbohydrate-binding module
CE: carbohydrate esterase
COGs: clusters of orthologous groups
eggNOG: non-supervised orthologous groups
EGTs: environmental gene tags
GH: glycoside hydrolase
GT: glycosyltransferase
HMMs: hidden markov models
IMG/M: integrated microbial genomes and metagenomes
KEGG: kyoto encyclopedia of genes and genomes
MG-RAST: metagenomics RAST
ORF: open reading frame
OTU: operational taxonomic unit
PL: polysaccharide lyases
RDP: ribosomal database project
RSA: rice straw-adapted
SSU: small subunit
