# Supplementary material for: Metagenomic analysis of microbial consortia enriched from compost: new insights into the role of Actinobacteria in lignocellulose decomposition
Source: Biotechnol Biofuels. 2016 Jan 29;9:22. doi: 10.1186/s13068-016-0440-2 (PMC4731972; doi:10.1186/s13068-016-0440-2)
Supplement: Supplementary file 1 — 10.1186/s13068-016-0440-2 Comparison of the content of pre- and post- enrichment rice straw and its components (34 k). [file 13068_2016_440_MOESM1_ESM.doc]

**Additional file 1: Table S1** Comparison of the content of pre- and post- enrichment rice straw and its components

|  | **Rice straw (g)** | **Cellulose (g)** | **Hemicellulose (g)** | **Lignin (g)** |
| --- | --- | --- | --- | --- |
| Pre-enrichment | 14.81 | 5.23 | 3.43 | 1.80 |
| Post-enrichment | 10.15 | 3.39 | 1.60 | 1.48 |
