# Supplementary figures and images for: Metagenomic analysis of microbial consortia enriched from compost: new insights into the role of Actinobacteria in lignocellulose decomposition
Source: Biotechnol Biofuels. 2016 Jan 29;9:22. doi: 10.1186/s13068-016-0440-2 (PMC4731972; doi:10.1186/s13068-016-0440-2)

**Post-enrichment**

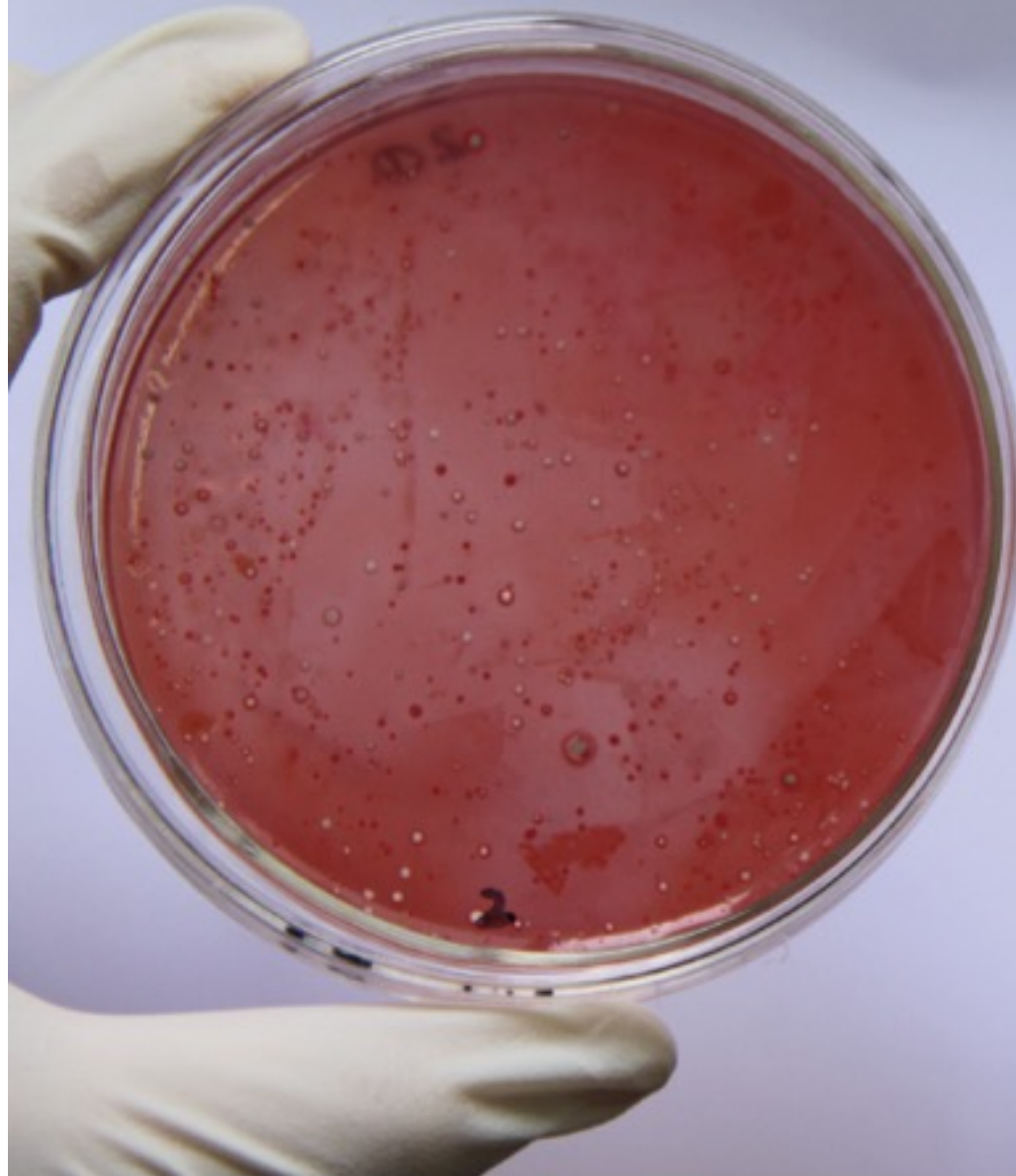

**Pre-enrichment**

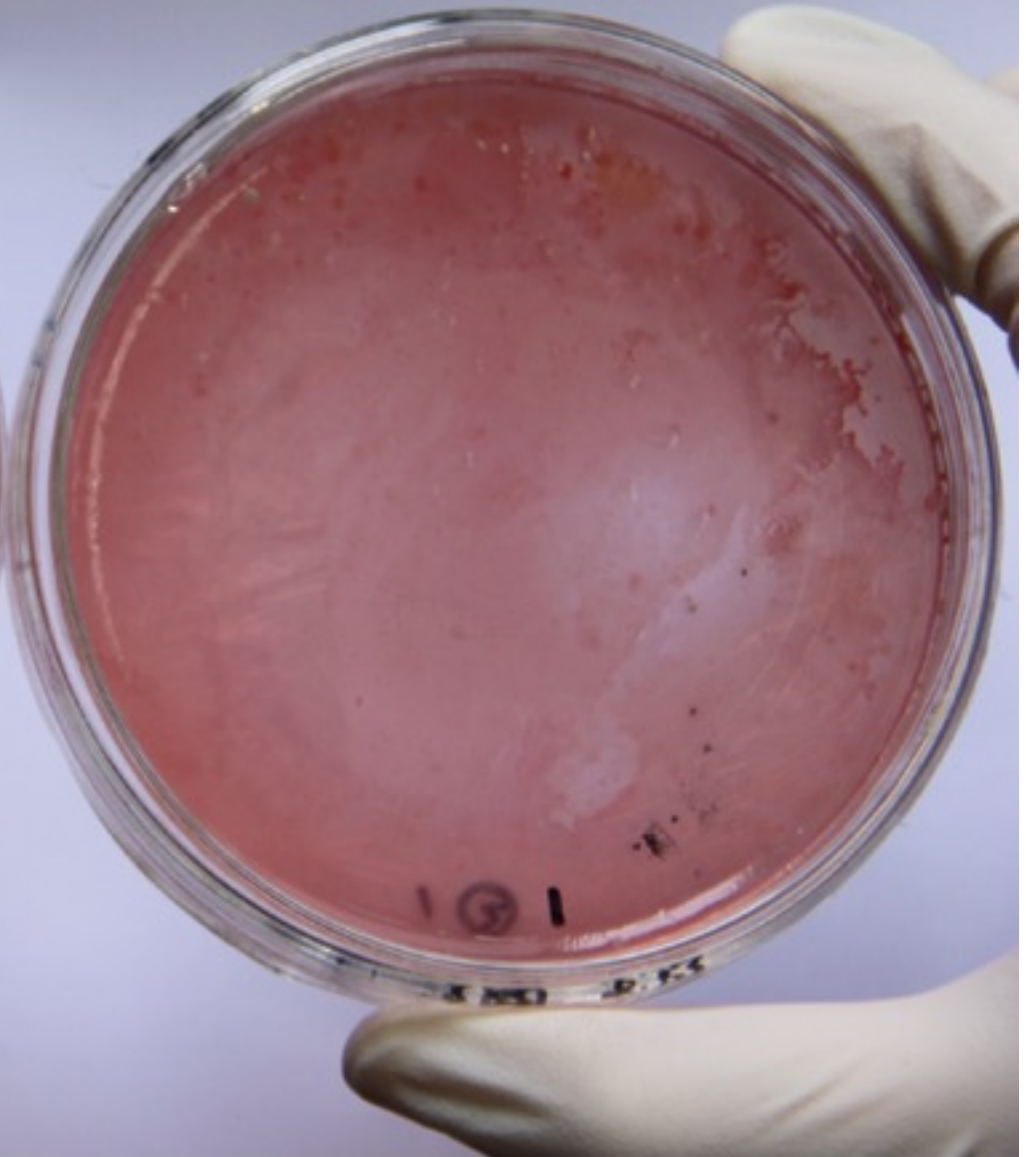

Supplement: Supplementary file 2 — 10.1186/s13068-016-0440-2 Cultures grown on Congo red agar plates from post-enrichment rice straw (left) and pre-enrichment rice straw (right). About 1 g sample was added into 100 mL of sterile water and shaken at 150 rpm for 1 h. Then 100 μL of the resultant solution was inoculated on Congo red agar plates at 28 °C for 7 days. The Congo red agar medium contained K2HPO4 (0.5 g), MgSO4 (0.25 g), CMC-Na (1.88 g), yeast extract (1.0 g), Congo-red (0.4 g), agar (14.0 g), gelatin (2.0 g), pH 7.0 (per liter) (89 k). [file 13068_2016_440_MOESM2_ESM.pdf]

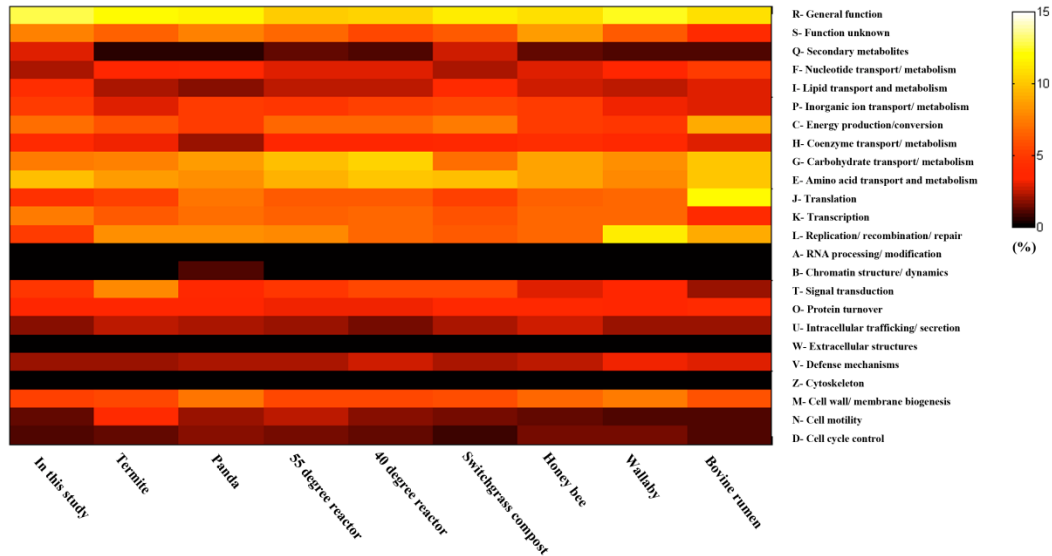

Supplement: Supplementary file 5 — 10.1186/s13068-016-0440-2 Metabolic clustering of RSA consortia, termite, panda, reactors, switchgrass-adapted compost, honey bee, wallaby, and bovine rumen metagenomes. The different color represents relative percentage of the metabolic classes within each sample (112 k). [file 13068_2016_440_MOESM5_ESM.pdf]

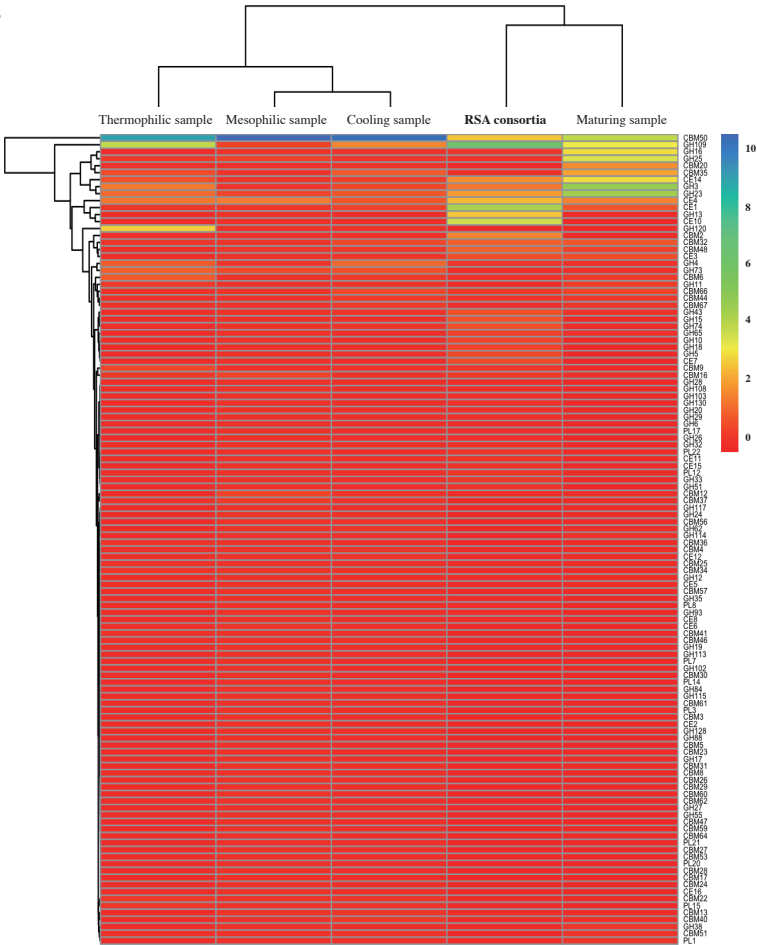

Supplement: Supplementary file 7 — 10.1186/s13068-016-0440-2 A heat map cluster representing CAZyme profiles in the RSA consortia Metagenome and four typical sample Metatranscriptomes (including samples from the mesophilic stage, thermophilic stage, cooling stage and maturing stage of the composting process; unpublished data). The relative abundances of gene and transcript in Metegenome and Metatranscriptome, respectively, were standardized by z-score transformation (236 k). [file 13068_2016_440_MOESM7_ESM.pdf]

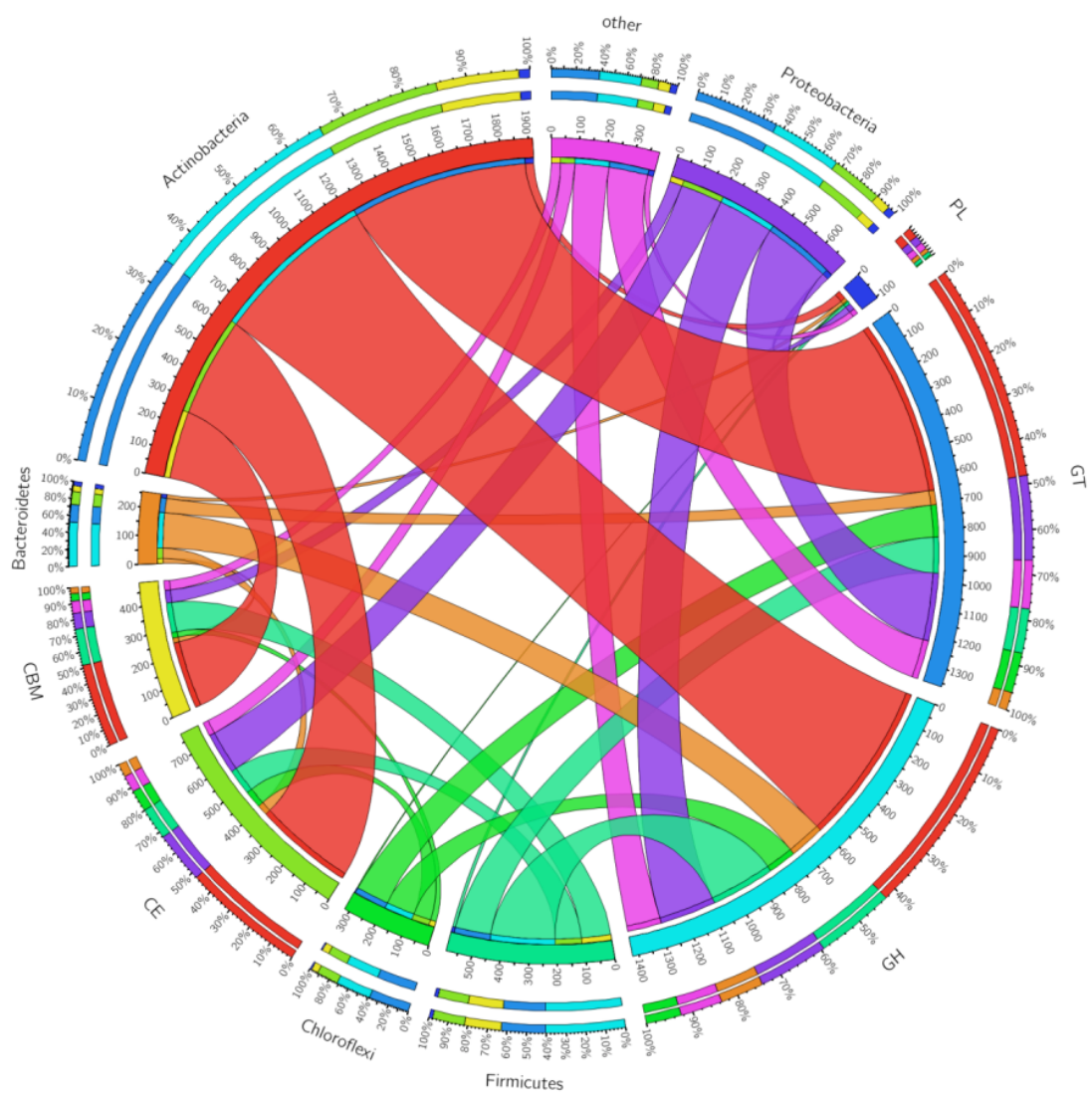

Supplement: Supplementary file 8 — 10.1186/s13068-016-0440-2 Phylogenetic distributions of carbohydrate-active enzymes at the phylum level in the RSA consortia. The data were visualized via Circos software (427 k). [file 13068_2016_440_MOESM8_ESM.pdf]
