# Supplementary material for: Metagenomic analysis of microbial consortia enriched from compost: new insights into the role of Actinobacteria in lignocellulose decomposition
Source: Biotechnol Biofuels. 2016 Jan 29;9:22. doi: 10.1186/s13068-016-0440-2 (PMC4731972; doi:10.1186/s13068-016-0440-2)
Supplement: Supplementary file 3 — 10.1186/s13068-016-0440-2 Summary of illumina GA reads and ORF prediction (33 k). [file 13068_2016_440_MOESM3_ESM.doc]

**Additional file 3: Table S2 Summary of Illumina GA reads and ORF prediction**

| **Sample ID** | **Raw reads** | **Clean reads** | **Clean reads**  **rate (%)** | **ORFs** | **Total**  **length** | **Average length** | **Complete ORFs** | **Fragmental ORFs** |
| --- | --- | --- | --- | --- | --- | --- | --- | --- |
| RS | 60,000,000 | 59,627,950 | 99.38 | 174,022 | 114,293,784 | 657 | 69,017 | 105,005 |
