# Supplementary material for: Metagenomic analysis of microbial consortia enriched from compost: new insights into the role of Actinobacteria in lignocellulose decomposition
Source: Biotechnol Biofuels. 2016 Jan 29;9:22. doi: 10.1186/s13068-016-0440-2 (PMC4731972; doi:10.1186/s13068-016-0440-2)
Supplement: Supplementary file 4 — 10.1186/s13068-016-0440-2 MetaPhlAn species abundances in the RSA consortia (41 k). [file 13068_2016_440_MOESM4_ESM.doc]

**Additional file 4: Table S3: MetaPhlAn species abundances** in the RSA consortia

| **Ranking** | **Species** | **Affiated Phylum** | **Estimated abundance (%)** |
| --- | --- | --- | --- |
| 1 | *Thermobispora bispora* | *Actinobacteria* | 76.22 |
| 2 | *Rhodothermus marinus* | *Bacteroidetes* | 14.14 |
| 3 | *Sphaerobacter thermophilus* | *Chloroflexi* | 5.84 |
| 4 | *Thermomonospora curvata* | *Actinobacteria* | 3.20 |
| 5 | *Symbiobacterium thermophilum* | *Firmicutes* | 0.16 |
| 6 | *Thermobifida fusca* | *Actinobacteria* | 0.14 |
| 7 | *Mycobacterium unclassified* | *Actinobacteria* | 0.08 |
| 8 | *Thermocrinis albus* | *Aquificae* | 0.04 |
| 9 | *Sulfurihydrogenibium azorense* | *Aquificae* | 0.01 |
| 10 | *Geobacillus thermodenitrificans* | *Firmicutes* | 0.01 |
| 11 | *Thermoproteus neutrophilus* | *Crenarchaeota* | 0.01 |
| 12 | *Geobacillus thermoglucosidasius* | *Firmicutes* | 0.01 |
| 13 | *Pyrobaculum islandicum* | *Crenarchaeota* | 0.01 |
