# Supplementary material for: Metagenomic analysis of microbial consortia enriched from compost: new insights into the role of Actinobacteria in lignocellulose decomposition
Source: Biotechnol Biofuels. 2016 Jan 29;9:22. doi: 10.1186/s13068-016-0440-2 (PMC4731972; doi:10.1186/s13068-016-0440-2)
Supplement: Supplementary file 6 — 10.1186/s13068-016-0440-2 Glycan metabolism COGs enriched in rice straw-adapted microbial consortia enriched from manure compost (89 k). [file 13068_2016_440_MOESM6_ESM.doc]

**Additional file 6: Table S4 Glycan degradation COGs enriched in rice straw-adapted microbial consortia enriched from manure compost**

| **Category** | **COG** | **Annotation** | **Total hits in consortia** |
| --- | --- | --- | --- |
| **General** | COG1070 | Sugar (pentulose and hexulose) kinases | 100 |
|  | COG0061 | Predicted sugar kinase | 43 |
|  | COG0366 | Glycosidases | 113 |
|  | COG1082 | Sugar phosphate isomerases/epimerases | 196 |
|  |  |  |  |
| **Arabinose** | COG3534 | Alpha-L-arabinofuranosidase | 38 |
|  | COG2160 | L-arabinose isomerase | 21 |
|  |  |  |  |
| **Fructose** | COG1621 | Beta-fructosidases (levanase/invertase) | 24 |
|  | COG0205 | 6-phosphofructokinase | 66 |
|  | COG1105 | Fructose-1-phosphate kinase and related fructose-6-phosphate kinase (PfkB) | 34 |
|  |  |  |  |
| **Fucose** | COG2407 | L-fucose isomerase and related proteins | 13 |
|  | COG4154 | Fucose dissimilation pathway protein FucU | 9 |
|  | COG3669 | Alpha-L-fucosidase | 17 |
|  |  |  |  |
| **Galactose** | COG1486 | Alpha-galactosidases/6-phospho-beta-glucosidases, family 4 of glycosyl hydrolases | 28 |
|  | COG3250 | Beta-galactosidase/beta-glucuronidase | 58 |
|  | COG3345 | Alpha-galactosidase | 14 |
|  | COG0153 | Galactokinase | 38 |
|  | COG2723 | Beta-glucosidase/6-phospho-beta-glucosidase/beta-galactosidase | 88 |
|  |  |  |  |
| **Glucuronose** | COG3661 | Alpha-glucuronidase | 16 |
|  | COG1904 | Glucuronate isomerase | 24 |
|  | COG3250 | Beta-galactosidase/beta-glucuronidase | 58 |
|  |  |  |  |
| **Glucosamine** | COG1820 | N-acetylglucosamine-6-phosphate deacetylase | 51 |
|  | COG0363 | 6-phosphogluconolactonase/Glucosamine-6-phosphate isomerase/deaminase | 42 |
|  |  |  |  |
| **Glucose** | COG3405 | Endoglucanase Y | 1 |
|  | COG3459 | Cellobiose phosphorylase | 15 |
|  | COG0297 | Glycogen synthase | 26 |
|  | COG0296 | 1,4-alpha-glucan branching enzyme | 63 |
|  | COG1523 | Type II secretory pathway, pullulanase PulA and related glycosidases | 56 |
|  | COG2723 | Beta-glucosidase/6-phospho-beta-glucosidase/beta-galactosidase | 88 |
|  | COG0166 | Glucose-6-phosphate isomerase | 55 |
|  | COG1501 | Alpha-glucosidases, family 31 of glycosyl hydrolases | 31 |
|  | COG1472 | Beta-glucosidase-related glycosidases | 99 |
|  |  |  |  |
| **Mannose** | COG1312 | D-mannonate dehydratase | 12 |
|  | COG0246 | Mannitol-1-phosphate/altronate dehydrogenases | 18 |
|  | COG1482 | Phosphomannose isomerase | 17 |
|  | COG1109 | Phosphomannomutase | 141 |
|  |  |  |  |
| **Rhamnose** | COG4806 | L-rhamnose isomerase | 2 |
|  |  |  |  |
| **Xylose** | COG3507 | Beta-xylosidase | 51 |
|  | COG3693 | Beta-1,4-xylanase | 52 |
|  |  |  |  |
| **Transferase** | COG0438 | Glycosyltransferase | 594 |
|  | COG1640 | 4-alpha-glucanotransferase | 26 |
|  |  |  |  |
| **Transport** | COG4209 | ABC-type polysaccharide transport system, permease component | 17 |
|  | COG0395 | ABC-type sugar transport system, permease component | 423 |
|  | COG2190 | Phosphotransferase system IIA components | 3 |
|  | COG1263 | Phosphotransferase system IIC components, glucose/maltose/N-acetylglucosamine-specific | 7 |
|  | COG1129 | ABC-type sugar transport system, ATPase component | 204 |
|  | COG1264 | Phosphotransferase system IIB components | 3 |
|  | COG1175 | ABC-type sugar transport systems, permease components | 435 |
|  | COG1299 | Phosphotransferase system, fructose-specific IIC component | 4 |
|  | COG1593 | TRAP-type C4-dicarboxylate transport system, large permease component | 118 |
|  | COG1638 | TRAP-type C4-dicarboxylate transport system, periplasmic component | 106 |
|  | COG1080 | Phosphoenolpyruvate-protein kinase (PTS system EI component in bacteria) | 46 |
|  | COG1445 | Phosphotransferase system fructose-specific component IIB | 1 |
|  | COG1653 | ABC-type sugar transport system, periplasmic component | 795 |
|  | COG1879 | ABC-type sugar transport system, periplasmic component | 141 |
|  | COG2814 | Arabinose efflux permease | 714 |
|  | COG1925 | Phosphotransferase system, HPr-related proteins | 27 |
|  | COG1762 | Phosphotransferase system mannitol/fructose-specific IIA domain (Ntr-type) | 23 |
