# Supplementary material for: Metagenomic analysis of microbial consortia enriched from compost: new insights into the role of Actinobacteria in lignocellulose decomposition
Source: Biotechnol Biofuels. 2016 Jan 29;9:22. doi: 10.1186/s13068-016-0440-2 (PMC4731972; doi:10.1186/s13068-016-0440-2)
Supplement: Supplementary file 9 — 10.1186/s13068-016-0440-2 Summary of carbohydrate-active enzymes found in the rice straw-adapted consortia enriched from manure compost (262 k). [file 13068_2016_440_MOESM9_ESM.doc]

**Additional file 9: Table S5 Summary of carbohydrate-active enzymes found in the rice straw-adapted consortia enriched from manure compost**

| CAZy family | Pfam HMM name | Pfam accession | Pfam description | Known activities | Rice straw - adapted community | |
| --- | --- | --- | --- | --- | --- | --- |
| Glycoside hydrolase catalytic domains | | | | | | |
| GH1 | Glyco_hydro_1 | PF00232 | Glycosyl hydrolase family 1 | β-Glucosidase, β-galactosidase, β-mannosidase, others | 63 | |
| GH2 | Glyco_hydro_2_C | PF00703 | Glycosyl hydrolase family 2, TIM barallel domain | β-Galactosidase, β-mannosidase, others | 27 | |
| GH3 | Glyco_hydro_3 | PF00933 | Glycosyl hydrolase family 3 N terminal domain | β-1,4-Glucosidase, β-1,4-xylosidase, β-1,3-glucosidase,α-larabinofuranosidase, others | 83 | |
| GH4 | Glyco_hydro_4 | PF02056 | Family 4 glycosyl hydrolase | α-Glucosidase, a-galactosidase, α-glucuronidase, others | 21 | |
| GH5 | Cellulase | PF00150 | Cellulase (glycosyl hydrolase family 5) | Cellulase, β-1,4-endoglucanase, β-1,3-glucosidase,β-1,4-endoxylanase, β-1,4-endomannanase, others | 40 | |
| GH6 | Glyco_hydro_6 | PF01341 | Glycosyl hydrolase family 6 | Endoglucanase, cellobiohydrolase | 13 | |
| GH9 | Glyco_hydro_9 | PF00759 | Glycosyl hydrolase family 9 | Endoglucanase, cellobiohydrolase, β-glucosidase | 19 | |
| GH10 | Glyco_hydro_10 | PF00331 | Glycosyl hydrolase family 10 | Xylanase, β-1,3-endoxylanase | 37 | |
| GH11 | Glyco_hydro_11 | PF00457 | Glycosyl hydrolase family 11 | Xylanase | 8 | |
| GH12 | Glyco_hydro_12 | PF01670 | Glycosyl hydrolase family 12 | Endoglucanase, β-1,3-1,4-glucanase, xyloglucan  hydrolase | 7 | |
| GH13 | Alpha amylase | PF00128 | Alpha amylase, catalytic domain | α-Amylase, catalytic domain, and related enzymes | 145 | |
| GH15 | Glyco_hydro_15 | PF00723 | Glycosyl hydrolase family 15 | Glucoamylase, glucodextranase | 51 | |
| GH16 | Glyco_hydro_16 | PF00722 | Glycosyl hydrolase family 16 | β-1,3(4)-Endoglucanase, others | 24 | |
| GH17 | Glyco_hydro_17 | PF00322 | Glycosyl hydrolase family 17 | Glucan endo-1,3-β-glucosidase, glucan 1,3-β-  glucosidase, licheninase, β-1,3-glucan  transglycosidase | 1 | |
| GH18 | Glyco_hydro_18 | PF00704 | Glycosyl hydrolase family 18 | Chitinase, endo-β-N-acetylglucosaminidase, noncatalytic proteins | 35 | |
| GH19 | Glyco_hydro_19 | PF00182 | Chitinase class I | Chitinase | 4 | |
| GH20 | Glyco_hydro_20 | PF00728 | Glycosyl hydrolase family 20 | β-Hexosaminidase, lacto-N-biosidase | 15 | |
| GH23 | SLT | PF01464 | Transglycosylase SLT domain | G-type lysozyme, peptidoglycan lytic transglycosylase | 113 | |
| GH24 | Phage_lysozyme | PF00959 | Phage lysozyme | Lysozyme | 1 | |
| GH25 | Glyco_hydro_25 | PF01183 | Glycosyl hydrolase family 25 | Lysozyme | 19 | |
| GH26 | Glyco_hydro_26 | PF02156 | Glycosyl hydrolase family 26 | Mannanase, β-1,3-xylanase | 14 | |
| GH27 | Melibiase | PF02065 | Melibiase | α-Galactosidase, α-N-acetylgalactosaminidase,  isomalto-dextranase | 1 | |
| GH28 | Glyco_hydro_28 | PF00295 | Glycosyl hydrolase family 28 | Polygalacturonase, rhamnogalacturonase, others | 11 | |
| GH29 | Alpha_L_fucos | PF01120 | Alpha-L-fucosidase | α-L-fucosidase | 15 | |
| GH30 | Glyco_hydro_30 | PF02055 | O-Glycosyl hydrolase family 30 | Glucosylceramidase, β-1,6-glucanase, β-xylosidase | 6 | |
| GH31 | Glyco_hydro_31 | PF01055 | Glycosyl hydrolases family 31 | α-Glucosidase, α-xylosidase, others | 19 | |
| GH32 | Glyco_hydro_32N | PF00251 | Glycosyl hydrolases family 32 N terminal | Levanase, invertase, others | 14 | |
| GH33 | BNR | PF02012 | BNR/Asp-box repeat | Sialidase, neuraminidase, trans-sialidase | 25 | |
| GH35 | Glyco_hydro_35 | PF01301 | Glycosyl hydrolases family 35 | β-Galactosidase | 7 | |
| GH36 | Blast search |  |  | α-Galactosidase, α-N-acetylgalactosaminidase,  stachyose synthase, raffinose synthase | 8 | |
| GH37 | Trehalase | PF01204 | Trehalase | α,α-trehalase | 3 | |
| GH38 | Glyco_hydro_38 | PF01074 | Glycosyl hydrolases family 38 N-terminal  domain | α-Mannosidase | 13 | |
| GH39 | Glyco_hydro_39 | PF01229 | Glycosyl hydrolases family 39 | β-Xylosidase, α-L-iduronidase | 32 | |
| GH42 | Glyco_hydro_42 | PF02449 | Beta-galactosidase | β-Galactosidase | 14 | |
| GH43 | Glyco_hydro_43 | PF04616 | Glycosyl hydrolases family 43 | β-Xylosidase, β-1,3-xylosidase, α-L-arabinofuranosidase, arabinanase, others | 51 | |
| GH44 | Glyco_hydro_44 | PF12891 |  | Endoglucanase, xyloglucanase | 1 | |
| GH47 | Glyco_hydro_47 | PF01532 | Glycosyl hydrolases family 47 | Α-mannosidase | 1 | |
| GH48 | Glyco_hydro_48 | PF02011 | Glycosyl hydrolases family 48 | Endoglucanase, chitinase, cellobiohydrolases, endoprocessive cellulases | 5 | |
| GH50 | Blast search |  |  | β-agarase | 1 | |
| GH51 | No Pfam | No Pfam |  | α-L-Arabinofuranosidase, endoglucanase | 24 | |
| GH52 | Glyco_hydro_52 | PF03512 | Glycosyl hydrolases family 52 | β-Xylosidase | 3 | |
| GH53 | Glyco_hydro_53 | PF07745 | Glycosyl hydrolases family 53 | Endo-1,4-β-galactanase | 6 | |
| GH55 | Blast search |  |  | Exo-1,3-glucanase, endo-1,3-glucanase | 1 | |
| GH57 | Glyco_hydro_57 | PF03065 | Glycosyl hydrolase family 57 | α-Amylase, 4-α-glucanotransferase, α-galactosidase, amylopullulanase | 20 | |
| GH59 | Glyco_hydro_59 | PF02057 | Glycosyl hydrolase family 59 | Galactocerebrosidase | 2 | |
| GH62 | Glyco_hydro_62 | PF03664 | Glycosyl hydrolase family 62 | α-L-arabinofuranosidase | 6 | |
| GH63 | Glyco_hydro_63 | PF03200 | Mannosyl oligosaccharide glucosidase | Processing α-glucosidase, α-1,3-glucosidase, α-glucosidase | 4 | |
| GH64 | Blast search |  |  | β-1,3-Glucanase | 4 | |
| GH65 | Glyco_hydro_65m | PF03632 | Glycosyl hydrolase family 65 central catalytic  domain | Trehalase, maltose phosphorylase, trehalose phosphorylase | 34 | |
| GH66 | No Pfam | No Pfam |  | Cycloisomaltooligosaccharide glucanotransferase, dextranase | 1 | |
| GH67 | Glyco_hydro_67 M | PF07488 | Glycosyl hydrolase family 67 middle domain | α-Glucuronidase, others | 6 | |
| GH73 | Glucosaminidase | PF01832 | Mannosyl-glycoprotein endo-beta-Nacetylglucosaminidase | Peptidoglycan hydrolase with endo-β-N-acetylglucosaminidase specificity | 12 | |
| GH74 | Blast search |  |  | Endoglucanase, oligoxyloglucan reducing end-specific cellobiohydrolase, xyloglucanase | 48 | |
| GH76 | Glyco_hydro_76 | PF03663 | Glycosyl hydrolase family 76 | α-1,6-Mannanase | 16 | |
| GH77 | Glyco_hydro_77 | PF02446 | 4-Alpha-glucanotransferase | 4-α-Glucanotransferase, amylomaltase | 18 | |
| GH78 | Bac_rhamnosidase | PF05592 | Bacterial alpha-L-rhamnosidase | α-L-Rhamnosidase | 39 | |
| GH84 | NAGidase |  | Hyaluronidase | N-Acetyl β-glucosaminidase, hyaluronidase | 5 | |
| GH85 | Glyco_hydro_85 | PF03644 | Glycosyl hydrolase family 85 | Endo-β-N-acetylglucosaminidase | 2 | |
| GH87 | Blast search |  |  | Mycodextranase, α-1,3-glucanase | 3 | |
| GH88 | Glyco_hydro_88 | PF07470 | Glycosyl hydrolase family 88 | D-4,5 Unsaturated β-glucuronyl hydrolase | 3 | |
| GH92 | Glyco_hydro_92 | PF07971 | Glycosyl hydrolase family 92 | α-1,2-Mannosidase | 8 | |
| GH93 | Blast search |  |  | Exo-1,5-α-L-arabinanase | 8 | |
| GH94 | Blast search |  |  | Cellobiose phosphorylase, cellodextrin phosphorylase, chitobiose phosphorylase, cyclic β-1,2-glucan synthase | 1 | |
| GH95 | Blast search |  |  | α-1,2-L-Fucosidase, α-L-fucosidase | 22 | |
| GH99 | Blast search |  |  | Glycoprotein endo-α-1,2-mannosidase | 3 | |
| GH100 | invertase_neut | PF12899 | Plant neutral invertase | Alkaline and neutral invertase | 1 | |
| GH102 | MltA | PF03562 | MltA specific insert domain | Peptidoglycan lytic transglycosylase | 4 | |
| GH103 | Blast search |  |  | Peptidoglycan lytic transglycosylase | 16 | |
| GH105 | Glyco_hydro_88 | PF07470 | Glycosyl hydrolase family 88 | Unsaturated rhamnogalacturonyl hydrolase | 4 | |
| GH106 | Blast search |  |  | α-L-rhamnosidase | 6 | |
| GH108 | Glyco_hydro_108 | PF05838 |  | N-acetylmuramidase | 12 | |
| GH109 | Blast search |  |  | α-N-acetylgalactosaminidase | 318 | |
| GH110 | Blast search |  |  | α-Galactosidase, α-1,3-galactosidase | 1 | |
| GH113 | Blast search |  |  | β-Mannanase | 4 | |
| GH114 | Blast search |  |  | Endo-α-1,4-polygalactosaminidase | 4 | |
| GH115 | Blast search |  |  | Xylan α-1,2-glucuronidase, α-(4-O-methyl)-glucuronidase | 5 | |
| GH116 | Blast search |  |  | β-Glucosidase, β-glucosidase, β-xylosidase | 3 | |
| GH117 | Blast search |  |  | a-1,3-L-neoagarooligosaccharide hydrolase, a-1,3-L-neoagarobiase | 2 | |
| GH120 | Blast search |  |  | β-Xylosidase | 3 | |
| GH123 | Blast search |  |  | Glycosphingolipid β-N-acetylgalactosaminidase | 3 | |
| GH125 | Blast search |  |  | Exo-a-1,6-mannosidase | 4 | |
| GH127 | Blast search |  |  | β-L-arabinofuranosidase | 17 | |
| GH128 | Blast search |  |  | β-1,3-glucanase | 2 | |
| GH129 | Blast search |  |  | a-N-acetylgalactosaminidase | 3 | |
| GH130 | Blast search |  |  | 1-β-D-mannopyranosyl-4-D-glucopyranose:phosphate a-D-mannosyltransferase, β-1,4-mannooligosaccharide phosphorylase | 16 | |
|  |  |  |  |  |  |  |
| Carbohydrate binding domains | | | | | |  |
| CBM2 | CBM_2 | PF00553 | Cellulose binding domain | Cellulose binding domain | 94 |  |
| CBM3 | CBM_3 | PF00942 | Cellulose binding domain | Cellulose binding domain | 3 |  |
| CBM4 | CBM_4_9 | PF02018 | Carbohydrate binding domain | Carbohydrate binding domain | 6 |  |
| CBM5 | CBM_5_12 | PF02839 | Carbohydrate binding domain | Carbohydrate binding domain | 3 |  |
| CBM6 | CBM_6 | PF03422 | Carbohydrate binding module (fam. 6) | Carbohydrate binding module (family 6) | 19 |  |
| CBM8 | Blast search |  |  | Cellulose binding domain | 1 |  |
| CBM9 | CBM_4_9 | PF02018 | Carbohydrate binding domain | Carbohydrate binding domain | 22 |  |
| CBM12 | CBM_5_12 | PF02839 | Carbohydrate binding domain | Carbohydrate binding domain | 9 | |
| CBM13 | Ricin_B_lectin | PF00652 | Ricin-type beta-trefoil lectin domain | Ricin-type beta-trefoil lectin domain | 14 | |
| CBM16 | CBM_4_9 | PF02018 | Carbohydrate binding domain | Carbohydrate binding domain | 26 | |
| CBM20 | CBM_20 | PF00686 | Starch binding domain | Starch binding domain | 5 | |
| CBM22 | CBM_4_9 | PF02018 | Carbohydrate binding domain | Carbohydrate binding domain | 12 | |
| CBM23 | Blast search |  |  | Mannan binding domain | 2 | |
| CBM25 | CBM_25 | PF03423 | Carbohydrate binding domain (fam 25) | Carbohydrate binding domain (family 25) | 8 | |
| CBM30 | Blast search |  |  | Cellulose binding domain | 4 | |
| CBM32 | F5_F8_type_C | PF00754 | F5/8 type C domain | F5/8 type C domain | 63 | |
| CBM33 | Chitin_bind_3 | PF03067 | Chitin binding domain | Chitin binding domain | 18 | |
| CBM34 | Alpha-amylase_N | PF02903 | Alpha amylase, N-terminal ig-like domain | Alpha amylase, N-terminal ig-like domain | 5 | |
| CBM35 | Blast search |  |  | Xylan, mannan and beta-galactan binding domain | 17 | |
| CBM37 | Blast search |  |  | Broad binding specificity | 3 | |
| CBM40 | Sialidase |  |  | Sialic acid binding domain | 10 | |
| CBM44 | Blast search |  |  | Cellulose and xyloglucan binding domain | 24 | |
| CBM46 | Blast search |  |  | Cellulose binding domain | 1 | |
| CBM47 | F5_F8_type_C | PF00754 | F5/8 type C domain | F5/8 type C domain | 1 | |
| CBM48 | CBM_48 | PF02922 | Carbohydrate-binding module 48 (Isoamylase  N-terminal domain) | Carbohydrate-binding module 48 (Isoamylase N-terminal domain) | 65 | |
| CBM50 | LysM | PF01476 | LysM domain | LysM | 143 | |
| CBM51 | NPCBM | PF10633 | NPCBM/NEW2 domain | NPCBM-associated, NEW3 domain of alpha-galactosidase | 4 | |
| CBM57 | Blast search |  |  | Domains attached to various glycosidases | 6 | |
| CBM59 | Blast search |  |  | Mannan, xylan, and cellulose binding domain | 1 | |
| CBM61 | Blast search |  |  | beta-1,4-galactan binding domain | 3 | |
| CBM66 | Blast search |  |  | Exo-acting b-fructosidase SacC | 27 | |
| CBM67 | Blast search |  |  | L-rhamnose binding domain | 14 | |
| Polysaccharide lyases | | | | | | |
| PL1 | Pec_lyase_C | PF00544 | Pectate lyase | Pectate lyase | 8 | |
| PL2 | Blast search |  | Polysaccharide Lyase Family 2 | Pectate lyase, exo-polygalacturonate lyase | 1 | |
| PL3 | Pectate_lyase | PF03211 | Pectate lyase | Pectate lyase superfamily protein | 4 | |
| PL6 | Blast search |  |  | Alginate lyase, chondroitinase B | 1 | |
| PL7 | Blast search |  |  | Alginate lyase, α-L-guluronate lyase | 4 | |
| PL8 | Lyase_8 | PF02278 | Polysaccharide Lyase Family 8 | Polysaccharide lyase family 8, super-sandwich domain | 7 | |
| PL9 | Blast search |  | Polysaccharide Lyase Family 9 | Pectate lyase, exopolygalacturonate lyase | 15 | |
| PL10 | Blast search |  | Polysaccharide Lyase Family10 | Pectate lyase | 8 | |
| PL11 | Blast search |  | Polysaccharide Lyase Family 11 | Rhamnogalacturonan lyase | 10 | |
| PL12 | Blast search |  | Polysaccharide Lyase Family 12 | Heparin-sulfate lyase | 25 | |
| PL14 | Blast search |  | Polysaccharide Lyase Family 14 | Alginate lyase, polysaccharide lyase acting on  glucuronic acid | 5 | |
| PL15 | Blast search |  | Polysaccharide Lyase Family 15 | Oligo-alginate lyase | 11 | |
| PL17 | Blast search |  | Polysaccharide Lyase Family 17 | Alginate lyase | 14 | |
| PL22 | Blast search |  | Polysaccharide Lyase Family 22 | Oligogalacturonate lyase / oligogalacturonide lyase | 21 | |
| Carbohydrate esterases | | | | | | |
| CE1 | Blast search |  | Carbohydrate Esterase Family 1 | Acetyl xylan esterase, cinnamoyl esterase, feruloyl esterase | 223 | |
| CE2 | Blast search |  | Carbohydrate Esterase Family 2 | Acetyl xylan esterase | 3 | |
| CE3 | Blast search |  | Carbohydrate Esterase Family 3 | Acetyl xylan esterase | 46 | |
| CE4 | Polysacc_deac_1 | PF01522 | Polysaccharide deacetylase | Acetyl xylan esterase, chitin deacetylase,  chitooligosaccharide deacetylase | 134 | |
| CE5 | Blast search |  | Carbohydrate Esterase Family 5 | Acetyl xylan esterase, cutinase | 5 | |
| CE6 | Blast search |  | Carbohydrate Esterase Family 6 | Acetyl xylan esterase | 2 | |
| CE7 | Blast search |  | Carbohydrate Esterase Family 7 | Acetyl xylan esterase, cephalosporin-C deacetylase | 44 | |
| CE8 | Pectinesterase | PF01095 | Pectinesterase | Pectinesterase | 8 | |
| CE9 | Blast search |  | Carbohydrate Esterase Family 9 | N-acetylglucosamine 6-phosphate deacetylase, Nacetylgalactosamine-6-phosphate deacetylase | 46 | |
| CE10 | Blast search |  | Carbohydrate Esterase Family 10 | Arylesterase, carboxyl esterase, acetylcholinesterase, cholinesterase, sterol esterase, brefeldin A esterase | 192 | |
| CE11 | LpxC | PF03331 | UDP-3-0-acyl N-acetylglucosamine deacetylase | UDP-3-0-acyl N-acetylglucosamine deacetylase | 20 | |
| CE12 | Blast search |  | Carbohydrate Esterase Family 12 | Pectin acetylesterase, rhamnogalacturonan  acetylesterase, acetyl xylan esterase | 4 | |
| CE14 | PIG-L | PF02585 | GlcNAc-PI de-N-acetylase | GlcNAc-PI de-N-acetylase | 102 | |
| CE15 | Blast search |  | Carbohydrate Esterase Family 15 | 4-O-methyl-glucuronoyl methylesterase | 20 | |
