# Supplementary material for: Metagenomic analysis of microbial consortia enriched from compost: new insights into the role of Actinobacteria in lignocellulose decomposition
Source: Biotechnol Biofuels. 2016 Jan 29;9:22. doi: 10.1186/s13068-016-0440-2 (PMC4731972; doi:10.1186/s13068-016-0440-2)
Supplement: Supplementary file 10 — 10.1186/s13068-016-0440-2 Summary of Auxiliary Activities (AA) families found in the rice straw-adapted consortia enriched from manure compost (39 k). [file 13068_2016_440_MOESM10_ESM.doc]

**Additional file 10: Table S6 Summary of Auxiliary Activities (AA) families found in the rice straw-adapted consortia enriched from manure compost**

| **Families** | **Known activities** | **EC number** | **Rice straw - adapted community** |
| --- | --- | --- | --- |
| AA2 | Class II peroxidase | EC 1.11.1.13  EC 1.11.1.14  EC 1.1.1.1.16 | 23 |
| AA3 | GMC oxidoreductase | EC 1.1.99.18  EC 1.1.3.7/1.1.3.4  EC 1.1.3.13  EC 1.1.3.10 | 56 |
| AA4 | Vanillyl alcohol oxidase | EC 1.1.3.38 | 36 |
| AA5 | Radical-copper oxidase | EC 1.1.3.-  EC 1.1.3.9 | 1 |
| AA6 | 1,4-Benzoquinone reductase | EC 1.6.5.6 | 38 |
| AA7 | Glucooligosaccharide oxidase | EC 1.1.3.- | 93 |
| AA10 | Lytic polysaccharide monooxygenase | EC 1.-.-.- | 17 |
