# Supplementary material for: Metagenomic analysis of microbial consortia enriched from compost: new insights into the role of Actinobacteria in lignocellulose decomposition
Source: Biotechnol Biofuels. 2016 Jan 29;9:22. doi: 10.1186/s13068-016-0440-2 (PMC4731972; doi:10.1186/s13068-016-0440-2)
Supplement: Supplementary file 11 — 10.1186/s13068-016-0440-2 Summary of de novo assembly results (37 k). [file 13068_2016_440_MOESM11_ESM.doc]

**Additional file 11: Table S7 Sum**mary of de novo assembly results

| **Sample ID** | **kmer** | **Contig number** | **Contig length (bp)** | **N50(bp)** | **N90(bp)** | **Max(bp)** | **Min(bp)** |
| --- | --- | --- | --- | --- | --- | --- | --- |
| RS | K35 | 88,228 | 136,140,003 | 2,138 | 638 | 231,238 | 500 |
| RS | K37 | 86,557 | 133,986,285 | 2,142 | 639 | 204,867 | 500 |
| RS | K39 | 84,709 | 131,980,102 | 2,160 | 641 | 178,085 | 500 |
| **RS** | **K41** | **83,237** | **129,551,542** | **2,153** | **641** | **312,042** | **500** |
| RS | K43 | 81,396 | 126,473,650 | 2,140 | 641 | 257,787 | 500 |
| RS | K45 | 79,536 | 123,420,105 | 2,129 | 643 | 254,762 | 500 |
| RS | K47 | 78,013 | 120,307,467 | 2,104 | 641 | 174,675 | 500 |
